# Unraveling the Role of Charge Patterning in the Micellar Structure of Sequence-Defined Amphiphilic Peptoid Oligomers by Molecular Dynamics Simulations

**DOI:** 10.1021/acs.macromol.2c00141

**Published:** 2022-06-14

**Authors:** Erin Tsai, Hishara Keshani Gallage Dona, Xinjie Tong, Pu Du, Brian Novak, Rolf David, Steven W. Rick, Donghui Zhang, Revati Kumar

**Affiliations:** † Department of Chemistry, Louisiana State University, Baton Rouge, Louisiana 70803, United States; ‡ Department of Mechanical and Industrial Engineering, Louisiana State University, Baton Rouge, Louisiana 70803, United States; § Department of Chemistry, University of New Orleans, New Orleans, Louisiana 70148, United States; ∥ Center for Computation and Technology, Louisiana State University, Baton Rouge, Louisiana 70803, United States

## Abstract

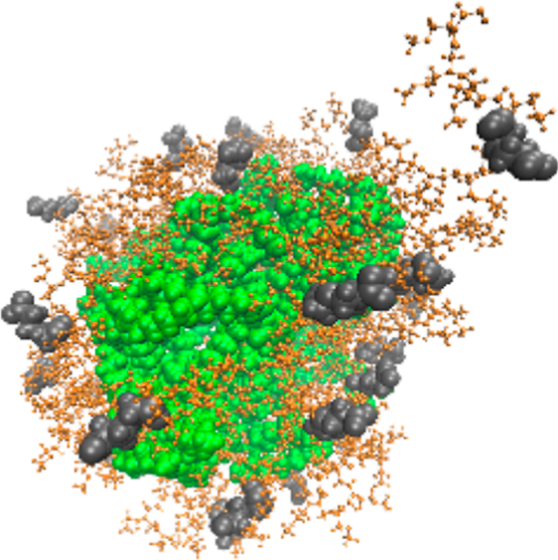

Electrostatic interactions play a significant role in regulating biological systems and have received increasing attention due to their usefulness in designing advanced stimulus-responsive materials. Polypeptoids are highly tunable N-substituted peptidomimetic polymers that lack backbone hydrogen bonding and chirality. Therefore, polypeptoids are suitable systems to study the effect of noncovalent interactions of substituents without complications of backbone intramolecular and intermolecular hydrogen bonding. In this study, all-atom molecular dynamics (MD) simulations were performed on micelles formed by a series of sequence-defined ionic polypeptoid block copolymers consisting of a hydrophobic segment and a hydrophilic segment in an aqueous solution. By combining the results from MD simulations and experimental small-angle neutron scattering data, further insights were gained into the internal structure of the formed polypeptoid micelles, which is not always directly accessible from experiments. In addition, information was gained into the physics of the noncovalent interactions responsible for the self-assembly of weakly charged polypeptoids in an aqueous solution. While the aggregation number is governed by electrostatic repulsion of the negatively charged carboxylate (COO^–^) substituents on the polypeptoid chain within the micelle, MD simulations indicate that the position of the charge on singly charged chains mediates the shape of the micelle through the charge–dipole interactions between the COO^–^ substituent and the surrounding water. Therefore, the polypeptoid micelles formed from the single-charged series offer the possibility for tailorable micelle shapes. In contrast, the polypeptoid micelles formed from the triple-charged series are characterized by more pronounced electrostatic repulsion that competes with more significant charge–sodium interactions, making it difficult to predict the shape of the micelles. This work has helped further develop design principles for the shape and structure of self-assembled micelles by controlling the position of charged moieties on the backbone of polypeptoid block copolymers.

## Introduction

Ubiquitous in nature and found commonly on the surface on many constituents of living cells, electrostatic interactions play a significant role in regulating biological systems.^1,2^ Biomacromolecules such as proteins, RNA, and DNA can be highly charged and use electrostatic interactions to maintain their functions and structure.^3−6^ Electrostatic interactions in molecular biology contribute to protein folding and stability, enzymatic recognition,^4^ protein–DNA interactions,^5^ protein–RNA binding^6,7^ and are implicated in various disease states involving intrinsically disordered proteins.^8,9^ Furthermore, electrostatic interactions on the nanoscale often dominate the physical forces that drive self-assembly and long-range spatial arrangement of many components in cells. In particular, while the hydrophobic effect is the primary driving force for protein folding, electrostatic interactions such as charge repulsion, charge attraction, or charge–dipole interactions (i.e., hydrogen bond formation) from charged amino acids allow proteins to assemble in complex, higher order structures.^10^ However, understanding the effect of charge location remains a substantial challenge for studying synthetic systems involving Coulombic interactions. Systems containing electrostatic interactions encounter unique challenges compared to nonionic systems due to phenomena such as osmotic swelling, electrostatic repulsion, and counterion condensation.^11−13^

Researchers have previously conducted systematic studies on the aggregation number and micellar size using ionic block copolymers, such as poly(styrene-*b*-acrylic acid),^14^ (dimethylamino)ethyl methacrylate,^15^ and poly(propylene imine-*b*-styrene) dendrimers,^16^ obtained using anionic polymerization or atom transfer radical polymerization. Although controlled living polymerization methods, such as ionic polymerizations, controlled radical polymerizations, and ring-opening polymerizations, have allowed for the synthesis of more complex multiblock copolymers with unique microstructures, these polymerization methods in general lack control over the exact arrangement of monomers along the polymer chain.^17^ As a result, several studies have employed synthetic polypeptide sequences to understand the self-assembly mechanisms of ionic block copolymers in an aqueous solution.^18−20^ While polypeptide sequences can be easily synthesized in a sequence-controlled manner, the intrinsic intramolecular hydrogen bonding and chirality of the backbone of polypeptides induce several secondary structures, such as α-helices and β-sheets.^21^ As a consequence, such secondary structures induced by hydrogen bonding and chirality in polypeptides further complicate the study of the sequence effect of changing the polymer’s substituents on their self-assembly structures.

Polypeptoids are synthetic, peptidomimetic polymers whose substituent group is attached to the nitrogen atom in the backbone instead of the α-carbon as in polypeptides.^22^ Furthermore, polypeptoids are attractive alternatives to overcome some of the limitations of therapeutic peptides^22−27^ and serve a wide range of applications in drug carriers,^28^ antifouling coatings,^29^ and antimicrobial agents.^30^ A consequence of N-substitution in polypeptoids versus substitution of the α-carbon as in polypeptides results in a lack of backbone hydrogen bonding and lack of chirality in polypeptoids. Therefore, the secondary structure in polypeptoids depends solely upon the chemical nature of the substituent group, eliminating the effect of intermolecular hydrogen bonding as found in β-sheets and intramolecular hydrogen bonding as found in α-helices of polypeptide folding.^25^ From a synthetic point of view, the controllable nature of a polypeptoid’s chemical sequence also allows for an excellent tunability of noncovalent secondary interactions, such as electrostatics, van der Waals interactions, hydrogen bonding, hydrophobicity, and hydrophilicity. For example, Kudirka et al. designed a polypeptoid block copolymer that can self-assemble into ultrathin, two-dimensional, highly ordered nanosheets.^31^ Their results demonstrated that intermolecular electrostatic interactions are the key factor in nanosheet formation. Sanii et al. also found that the hydrophobic air–water interface plays a crucial role in forming polypeptoid nanosheets and that the polypeptoid monolayer collapses into a colloidally stable free-floating bilayer.^32^ Noncovalent interactions can arise due to hydrophobic side groups, charged side groups, and hydrophilic neutral groups, which in turn can modulate the size and shape of micelles formed in aqueous solutions of polypeptoids.^33,34^ These properties of polypeptoids make them effective model systems to isolate the effect of secondary/noncovalent interactions of the substituent group on self-aggregation in a solution.

Investigations of the structural and dynamical properties of polypeptoid systems have been carried out using many theoretical and computational methods, including quantum-mechanical (QM) modeling, molecular dynamics (MD) simulations, Monte Carlo simulations, and coarse-grained modeling.^35−40^ Often coupling these simulations with enhanced sampling algorithms, such as parallel tempering^41^ or replica exchange molecular dynamics,^42^ umbrella sampling,^43^ and metadynamics,^44,45^ can significantly improve the sampling.^46−48^ Atomistic-level MD simulations on polypeptoids are challenging because of the comparatively long length and time scales that need to be simulated, making them time-consuming and costly. However, atomistic MD simulations can, in principle, provide a complete description of the molecular structure and an ample conformational space of these systems.^49,50^ Recently, researchers have parameterized atomistic level force fields designed explicitly for peptoids. Mirijanian et al. developed MFTOID, a CHARMM based force field, fitting to sarcosine dipeptoids in the vacuum and aqueous phases.^38^ However, the generated free energy Ramachandran plots indicated an overstabilized cis-helical conformation in the vacuum phase.^38,51^ Subsequently, Weiser and Santiso also presented a new CHARMM general force field (CGenFF), developed with the emphasis to accurately reproduce both the possible cis and trans isomerization of the polypeptoid backbone.^51^ Mukherjee et al. also reported that the general Amber force field GAFF could successfully predict a range of experimental structures for polypeptoid systems in an implicit solvent but worked poorly in an explicit solvent.^40^

Although the second generation of the general AMBER force field (GAFF2) was originally parameterized for generic organic molecules,^52,53^ in this work, the GAFF2 was validated by reproducing, reasonably well, both the cis and trans conformations of a simple sarcosine dipeptoid in the gas phase from quantum calculations (electronic structure calculations using density functional theory). GAFF2 was then used to perform atomistic MD simulations to study the self-assembly of singly and triply charged sequence-defined block copolymers in an aqueous solution, as reported by Sternhagen et al.^54^ In these experimental studies on a series of 25-mer polypeptoids with 5 consecutive hydrophobic monomers followed by 20 hydrophilic monomers, several of which were charged (with either one or three charged unit(s) per chain), the researchers found that the position of the charged group(s) has a significant effect on the aggregation number and the micelle size. One of the significant limitations of experimental work is that it is often difficult to give a detailed molecular picture of the micelle’s internal structure and to isolate the physical driving forces of micellar structural parameters. In this work, all-atom molecular dynamics (MD) simulations were used to elaborate on the atomistic structural properties of the micelles formed in an aqueous solution by probing the size, solvent-accessible surface area (SASA), asphericity, and shape of the micellar structure. In addition, the extent of the contributions of noncovalent interactions, such as charge solvation, sodium–carboxylate interactions, electrostatic repulsion, and the compactness of the micelles to these various structural properties was evaluated, which are not directly accessible through experiments.

## Methods

### Force Field Validation

**Figure 1:**
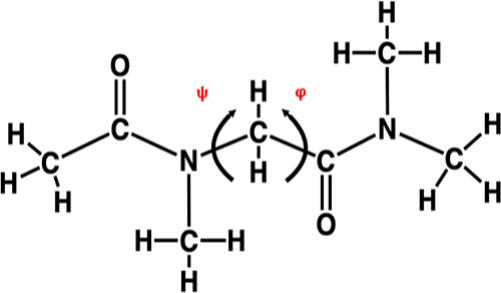
Chemical structure of sarcosine dipeptoid.

As mentioned previously, the quality of a simulation study depends on the accuracy of the force field, and hence validation of a force field is essential. Wang et al.^52^ created the original version of GAFF for atomistic MD simulations of small organic molecules compatible with existing AMBER force fields for proteins and nucleic acids. Subsequently, they developed a second generation of the original version of GAFF (GAFF2) with revised bonded parameters and optimized nonbonded parameters to more accurately reproduce the molecular geometries and the potential energy surfaces from quantum mechanical calculations of additional small organic molecules and to improve the transferability of GAFF to new systems.^52,53^ A sarcosine dipeptoid, a simple peptoid analog with a methyl side chain, was selected as the representative compound to test whether these GAFF2 parameters were appropriate for the studied polypeptoid systems (Figure 1). The potential energy profile of the sarcosine dipeptoid as a function of the two dihedral angles ψ and φ in the gas phase was calculated using the GAFF2 force field and validated from electronic structure scans. The partial charges of sarcosine were derived from the restrained electrostatic potential (RESP) method^55^ at the Hartree–Fock level of theory with the 6-31G* basis set using the Antechamber program.^56^ AMBER 16 was used to carry out these classical calculations.^56^ For electronic structure calculations of the dihedral angles’ potential energy profile, the energies and optimized geometries were obtained at the B3LYP/6-31G(d,p) level of theory. All electronic structure calculations were performed with Gaussian 09.^57^ The scans covered the entire 360° range of ψ, φ dihedral angles in increments of 20° to accurately capture the minima.

### Molecular Dynamics Simulation Details

**Figure 2:**
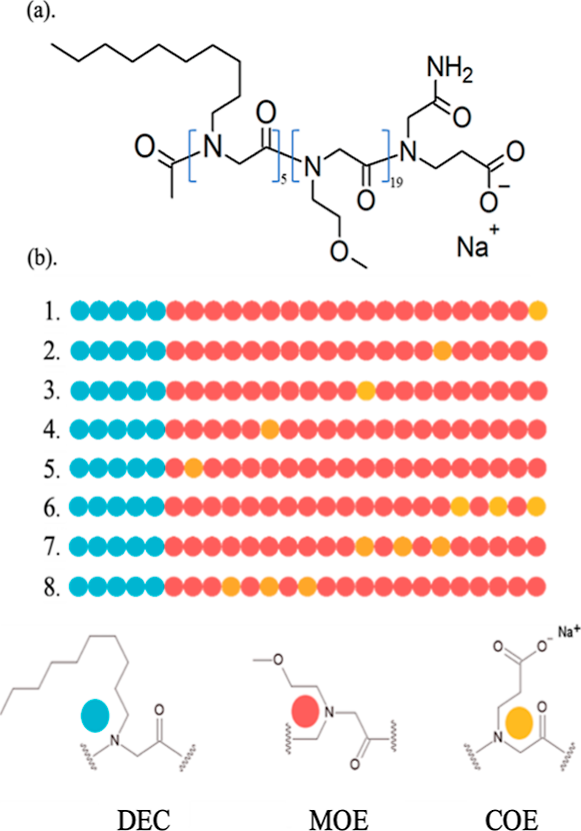
(a) Chemical structure of a representative sequence of the studied ionic peptoid block copolymers (chain 1) and (b) the sequence library of the singly charged (chain 1–5) and the triply charged series (chain 6–8), which are composed of three different monomers: DEC, MOE, and COE.

Singly and triply charged ionic polypeptoid block copolymers were synthesized and reported by Sternhagen et al.,^54^ as illustrated in Figure 2. The individual residues, *N*-decyl (DEC), *N*-methoxyethyl (MOE), and *N*-(2-carboxyethyl) (COE) from the sequences of the reported polypeptoid block copolymers were first constructed in Avogadro,^58^ and the initial structures of these polypeptoids were constructed by combining these residues. Topologies and parameters were generated by LEAP33 and antechamber modules in AMBER 16 (Figure S1).^56^ The GROMACS compatible topologies were generated through the ACPYPE command. All-atomistic simulations were performed in GROMACS 2018.3^59^ with the GAFF2 force field.^52,53^

For MD simulations in GROMACS, the Nose–Hoover thermostat^60,61^ was used for temperature coupling with a time constant of 0.4 ps, and the Parrinello–Rahman barostat^62^ was used for pressure coupling at 1 bar with a time constant of 2.5 ps. The LINCS algorithm^63^ was used for constraining covalent bonds involving hydrogen. Periodic boundary conditions were applied in all directions. The cutoff distance for van der Waals and short-range interactions was 0.8 nm. The particle mesh Ewald^64^ method was utilized for long-range Coulomb interactions.

### Micelle Formation

As a low concentration (1.0 wt %) system, it would be time-consuming for the individual polypeptoid chains to diffuse to form a micelle. Therefore, it was easier to form a micelle at a higher concentration and then add the resulting micelle to a diluted solution to reach a lower concentration (1.0 wt %). Micelle assembly was achieved by initially simulating at a higher concentration (4.0 wt %) until the micelle was obtained. The hydrophilic region of each surfactant chain was restrained to the outer shell of the micelle with the hydrophobic section near the center of the shell using PACKMOL,^65,66^ thereby bringing the individual chains closer to one another. For each micelle system, the number of polypeptoid chains was taken from the experiments by Sternhagen et al.^54^ The pre-equilibrated polypeptoid surfactants were hydrated with pre-equilibrated TIP3P water,^67^ and sodium ions were then added to maintain a neutralized system. The system was subsequently equilibrated for 5.0 ns to assemble the micelle. This method worked for most of the simulated polypeptoid systems, but for the micelles formed by the chains 3, 4, and 7, a few surfactant chains fragmented from the primary micelle containing most of the polymer chains after the mentioned equilibration. For the micelles formed by these three sequences, we employed the PULL code in GROMACS 2018.3^59^ that applies forces or constraints between the center of mass of the hydrophobic portion of the separated chain(s) and one of the polypeptoid chains within the main micellar structure. We used the constraint method of pulling with direction-periodic geometry at a rate of −0.003 ps/nm. Once the separated chain was pulled sufficiently close to the remaining polypeptoid chains, the system was equilibrated for 2.0 ns, followed by another 5.0 ns to ensure the stability of the revised micelle. The newly assembled micelle (excluding the water) was then extracted from the last snapshot, and the appropriate number of waters were added to dilute each micelle system to approximately 1.0 wt % (Table 1). The dimensions of the simulation box for the micellar system were dependent upon the number of calculated water molecules to obtain 1.0 wt % of polypeptoid surfactants for each system (see Table 1 for box dimensions for each case).

**Table 1:** Overview of the Polypeptoid Systems^a^

chain #	# surfactant (N)	# water molecules	concentration (wt %)	simulation box dimensions
1	28	516,703	1.01	250 Å × 250 Å × 250 Å
2	25	461,503	1.01	241 Å × 241 Å × 241 Å
3	23	434,591	1.01	234 Å × 234 Å × 234 Å
4	18	332,282	1.02	216 Å × 216 Å × 216 Å
5	13	239,960	1.01	194 Å × 194 Å × 194 Å
6	18	369,782	1.00	223 Å × 223 Å × 223 Å
7	17	315,291	1.00	212 Å × 212 Å × 212 Å
8	12	206,465	1.00	184 Å × 184 Å × 184 Å

a The number of chains, *N*, was informed from the experiments by Sternhagen et al.^54^

For each micelle, six different simulations were carried out. The assembled micellar system (configuration 1) was linearly heated from 300 to 350 K (to form configuration 2) and 400 K (to form configuration 3) in 3.0 ns and then slowly cooled down to 300 K at the rate of 1 K/ns to prepare three different starting configurations for the first three simulations. A constant number of atoms, pressure (1 bar), and temperature (*NPT*) were used with a time step of 2.0 fs for each polypeptoid system. Each starting configuration was equilibrated for 50 ns and followed by another 50 ns production run in the isothermal–isobaric (*NPT*) ensemble^62^ with the temperature at 300 K, an average pressure of 1 bar, and a time step of 2.0 fs. Three additional starting configurations were generated from the resulting snapshots of the first production run to further increase sampling by linearly heating each output configuration from 300 to 400 K in 3.0 ns and then slowly cooling back to 300 K at a rate of 1 K/ns (configurations 4, 5, 6). The generated snapshots from the new starting configurations for the second series of simulations were once again equilibrated for 50 ns, followed by another 50 ns production run in the isothermal–isobaric (*NPT*) ensemble with the temperature at 300 K with an average pressure of 1 bar and a time step of 2.0 fs. Hence, a total of 600 ns of data was obtained, of which 300 ns (the last 50 ns for each simulation) was used for analyses. A discussion of the choice of simulation length along with associated Figures S2 and S3 are given in the Supporting Information.

### Micelle Characterization

To determine the structural features of self-assembled micelles, the radius of gyration (*R*_g_) and the principal radii of gyration were calculated using the inertial tensor. The axis components correspond to the mass-weighted root-mean-square of the components orthogonal to each axis (eq 1)^68^
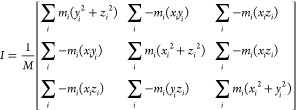
1where *m*_*i*_ is the mass, and *x*_i_, *y*_*i*_, and *z*_*i*_ are the coordinates of the *i*th atom, with the center of mass of the chain being the origin of the coordinate system. *M* is the total mass of the micelle. The eigenvalues denoted as λ_*i*_ (λ_1_ > λ_2_ > λ_3_) of the inertia tensor are related to *R*_g_ through the relationship *R*_g_^2^ = ∑*i*^3^λ_*i*_. The radius of gyration was also calculated using the scattering length weighted rather than the mass-weighted version of the tensor in eq 1 and is denoted as *R*_gb_.

### Small-Angle Neutron Scattering Intensity

Small-angle neutron scattering experiments (SANS) can be used to determine the *R*_g_ of the micelle, which in turn is an indicator of the size and compactness of the micelle and serves as one validation method for experimental work. *R*_g_ from the SANS experiments was determined from the micellar form factor *P*(*Q*), excluding the surrounding water. The total weighted radial distribution function (*g*(*r*)) can be calculated from the partial radial distribution functions of atoms α and β, *g*_αβ_(*r*), from the MD trajectories using the following equation, where *c*_α_is the number density, and *b*_α_ is the scattering length of atom α (eq 2)^69,70^
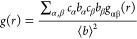
2

The radial distribution function is directly related via Fourier transform to the static structure factor of the system of interest (which, in turn, is directly proportional to the neutron scattering intensity)^71−73^ that can be determined using the following equation, where ρ is the density, and *Q* is the magnitude of the scattering vector
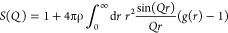
3

### Asphericity

The asphericity parameter measures the deviation from spherical symmetry and is defined by nonzero values from 0 to 1. Asphericity values that are closer to 0 indicate more spherical symmetry, while asphericity values closer to 1 indicate more deviation from the spherical symmetry (more aspherical). The asphericity parameter can then be derived from the inertia tensor using the following equation^74^
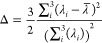
4where λ̅ is the mean of the three eigenvalues.

### Shape

Additional insights into the conformation of the micellar structure can be gained from analyzing the overall shape of the micellar structure (*S*)^74^
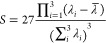
5

Negative values of *S* correspond to oblate shapes and positive values to prolate shapes. In the case of perfect oblate shapes, the shape of the structure appears flattened or disk-like (λ_1_ = λ_2_, λ_3_ = 0). In the case of perfect prolate shapes, the shape of the structure appears elongated and rodlike (λ_1_ ≠ 0, λ_2_ = λ_3_ = 0). The shape parameter, *S*, can take values between −0.25 and 2 (i.e. −0.25 ≤ *S* ≤ 2).^74^ Structures for which asphericity and shape are both 0 are spherical in nature.

### Solvent-Accessible Surface Area

The solvent-accessible surface area of each micelle was determined to assess the differences in solvation of the micelles formed by the charge placement at different positions along the polypeptoid chain.^75^ A brief description of the determination of the SASA is presented here. In general, a probe sphere molecule with a radius of 1.4 Å (mimicking the water molecule) is allowed to roll along the van der Waals surface of the micelle. The number of access points by the designated probe is multiplied by the surface area each point represents to calculate the SASA. The summation of the contact area gives the total solvent accessible surface area.^75^

## Results and Discussion

### Force Field Validation

**Figure 3:**
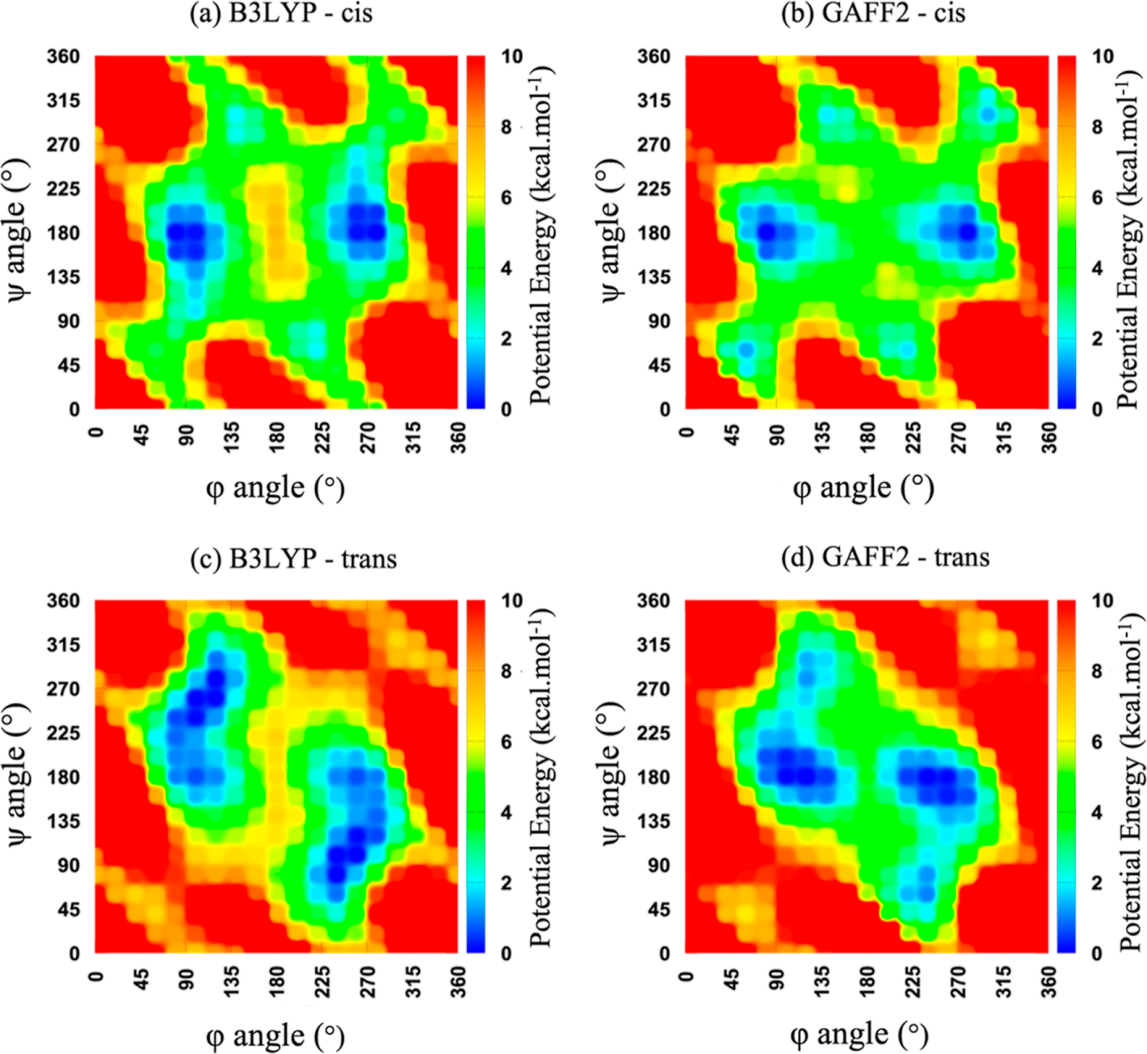
Potential energy (kcal/mol) surfaces of φ and ψ angles (degrees) for cis and trans sarcosine in the vacuum phase using (a,c) B3LYP/6-31G(d,p) and (b,d) GAFF2.

The 2D Ramachandran potential energy surface (PES) plots were calculated using GAFF2 parameters and compared to those generated using the B3LYP/6-31G(d,p) level of theory (Figure 3). For the cis conformation, the B3LYP PES has two global minima centered at (90, 180°) and (270, 180°), while the GAFF2 PES, similarly, has two global minima centered at (90, 180°) and (280, 180°). For the trans conformation, untuned GAFF2 predicts two different global minima. However, the energy difference is less than 2 kcal/mol between these minima. All these PES plots are center-symmetric because of the peptoid backbone achirality. While by no means quantitatively exact, GAFF2 does reproduce, reasonably well, the main features of the QM calculations for both cis and trans polypeptoid conformations.

### Micelle Characterization

**Figure 4:**
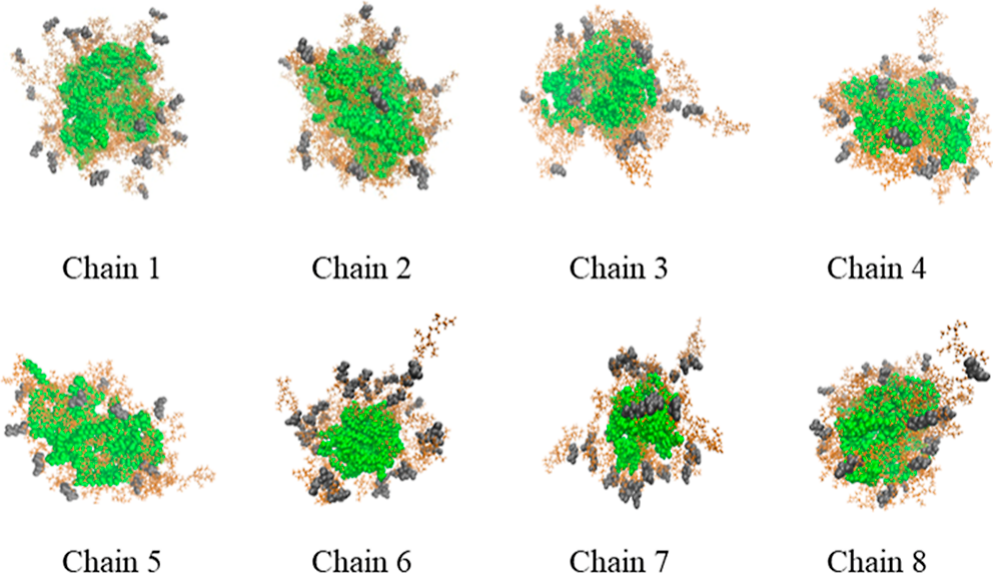
Representative snapshots of the micelles formed by the polypeptoid sequences (chain 1–chain 8) after the production run. Solid green spheres represent the hydrophobic DEC residues, orange ones represent the neutral MOE residues, and the grey ones represent the ionic COE residues.

The micelles under investigation remained stable throughout the entire simulation at a constant temperature and pressure with no change in the aggregation number. Each simulated micelle did not change significantly or adopt distinctly different structures. Representative snapshots of the micelles formed by the polypeptoid sequences after the production run are shown in Figure 4. The resulting snapshots demonstrate that the hydrophobic decyl units are compact and form the core of the micelle, while the hydrophilic methoxyethyl units are relatively less rigid. Furthermore, the ionic carboxylate substituents on the polypeptoid chains were more likely to orient themselves near the surface of the micelles, forming the micelles’ peripheral structure.

**Figure 5:**
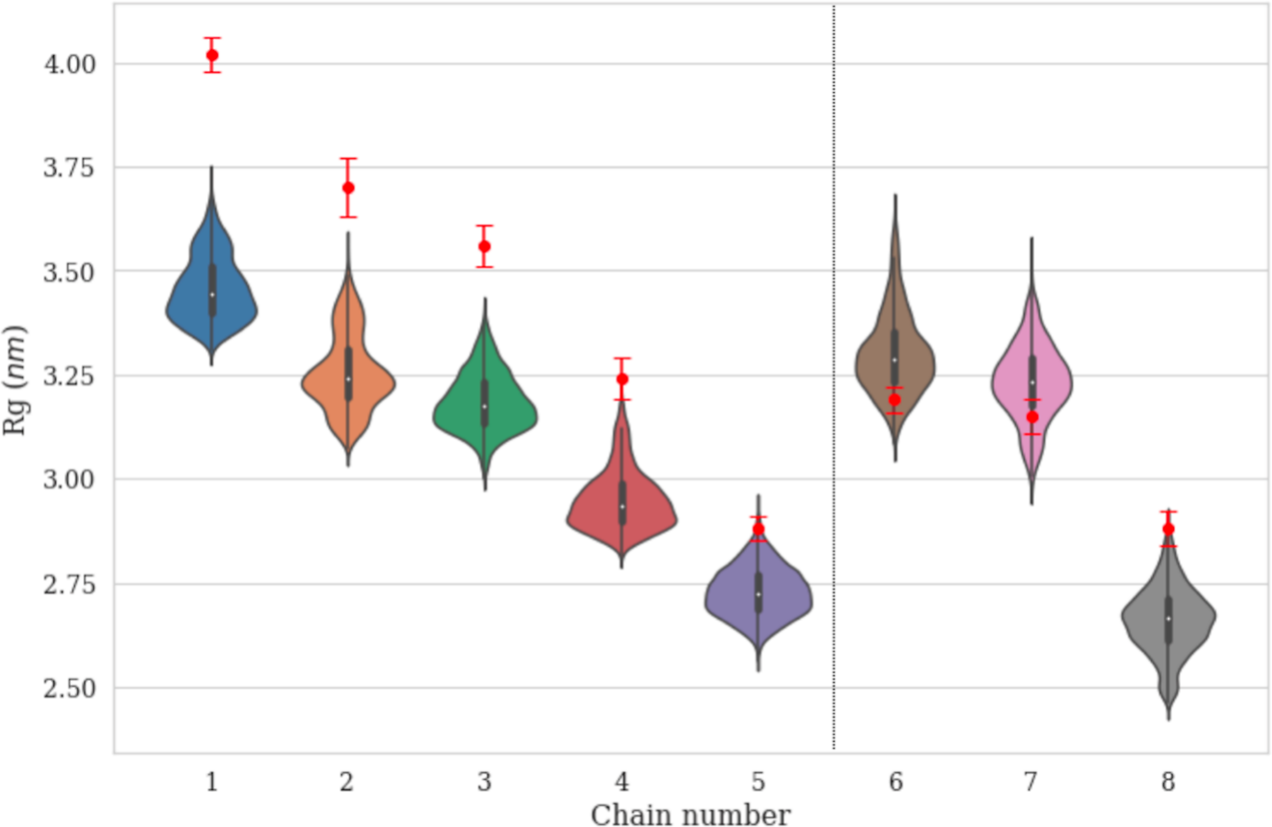
Violin plots of *R*_g_ of the micelles incorporating the water molecules within 0.35 nm (based on the oxygen atom of water) around the surfactants formed by chains 1 to 8. The corresponding experimental values of the average *R*_g_ obtained from SANS in D_2_O for each chain are plotted in red.^54^

*R*_g_, which is an indicator of the size and compactness of the micelles, was calculated from the simulation trajectories for each micelle. *R*_g_ of the micelles was computed from the MD trajectories without solvating water molecules and from the MD trajectories incorporating the water molecules within 0.35 nm (using the oxygen atom of water) around the surfactants. The distance employed for this calculation comes from the first minimum in the oxygen–oxygen radial distribution function of water, which has often been used to define solvation environments.^76,77^ The average *R*_g_ of the micelles formed by the singly charged series (chains 1 to 5) ranged from 2.8 to 3.5 nm and were found to be slightly higher than the average *R*_g_ range from 2.7 to 3.3 nm of the polypeptoid micelles formed by the triply charged series (chains 6 to 8) as illustrated by Table S1. In the self-assembled micelles formed by the chains 5 and 8 with aggregation numbers of *N* = 13 and *N* = 12, respectively, it was not surprising that the smaller aggregation numbers resulted in smaller *R*_g_ values compared to other polypeptoid micelle systems with *N* = 17–28 (Table S1). When the ionic monomer(s) are placed closer to the hydrophobic/hydrophilic junction, interchain electrostatic repulsion increases, resulting in a decrease in the number of individual polypeptoid chains that form the self-assembled micelles (aggregation number).^54^ This trend was consistent with the reported experimental results, and the calculated *R*_g_ was closer to the experimental *R*_g_ when taking coordinating water molecules into consideration (Figure 5, Table S1). The experimental *R*_g_ values are essentially weighted by scattering length rather than mass-weighted, and hence, the scattering-length-weighted radius of gyration, *R*_gb_, values from simulations were also calculated and tabulated in Table S1. These values are much closer to the experimental values. This provides some measure of validation of our simulations. The remaining analyses were carried out using the mass-weighted version of the inertial tensor. Violin plots of *R*_g_ can be used to visualize the distributions of *R*_g_ while providing additional information such as the median (white dot), interquartile range (rectangular box), and the lower/upper adjacent values (the outer tips), as shown in Figure 5. The Supporting Information provides an additional discussion of violin plots (Figure S4) in addition to the violin plots depicting *R*_g_ of the micelles formed by chains 1 to 8, excluding the water molecules in the calculation (Figures S5 and S6). These violin plots were plotted using the Seaborn library.^78^

**Figure 6:**
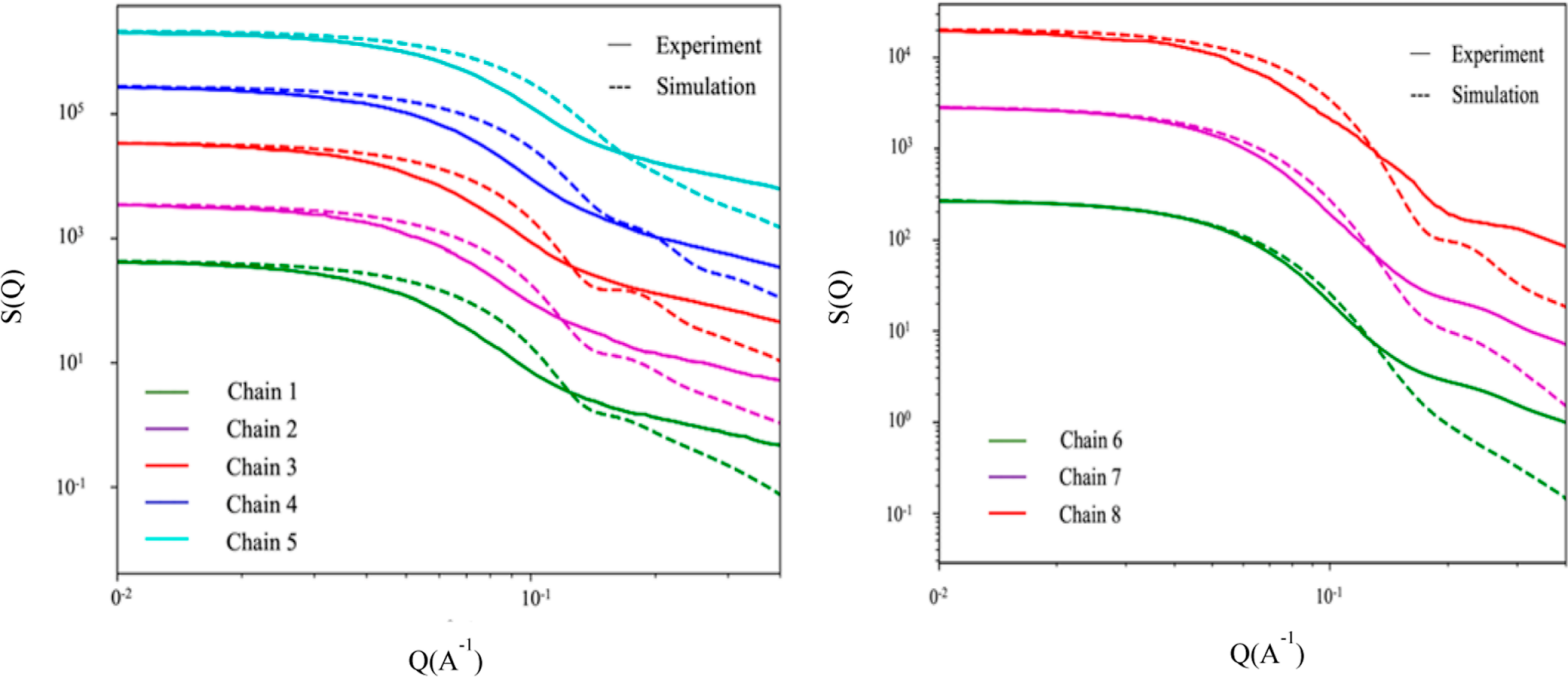
Static structure factors of the micelles formed by the singly charged series (chain 1 to 5) and the triply charged series (chain 6 to 8) computed from MD simulations in comparison with the experimental results obtained from SANS.^54^*S*(*Q*) is vertically scaled by a constant to align with the first data point in experiments for comparison and clarity. Note that water molecules were not included in the calculation of the static structure factor from simulations.

Experimentally, *R*_g_ was determined from Guinier’s analysis of the SANS experiments in D_2_O. The micellar static structure factor *S*(*Q*) obtained from SANS experiments and the calculated values (micelle only, no solvated water molecules) from the MD trajectories are illustrated in Figure 6. The low-Q region of the scattering curve represents a particle’s overall dimension. Comparing the experimental and simulated Guinier regions (region of the scattering curves with *Q* values ≤ 0.1 Å^–1^), the scattering curves computed from the simulations extended slightly toward higher *Q* values, indicating a slightly smaller overall size of the micelles than their experimental counterparts. However, it should be noted that the SANS experiments were performed on a 0.5 wt % concentration of the peptoid solution, whereas our simulations were performed at twice as high a concentration, namely at 1.0 wt %. Although *R*_g_ can provide quantitative information about the overall size of the micelles, it cannot give a detailed molecular picture of the internal structure of the micelle–water complex.

### Asphericity

**Figure 7:**
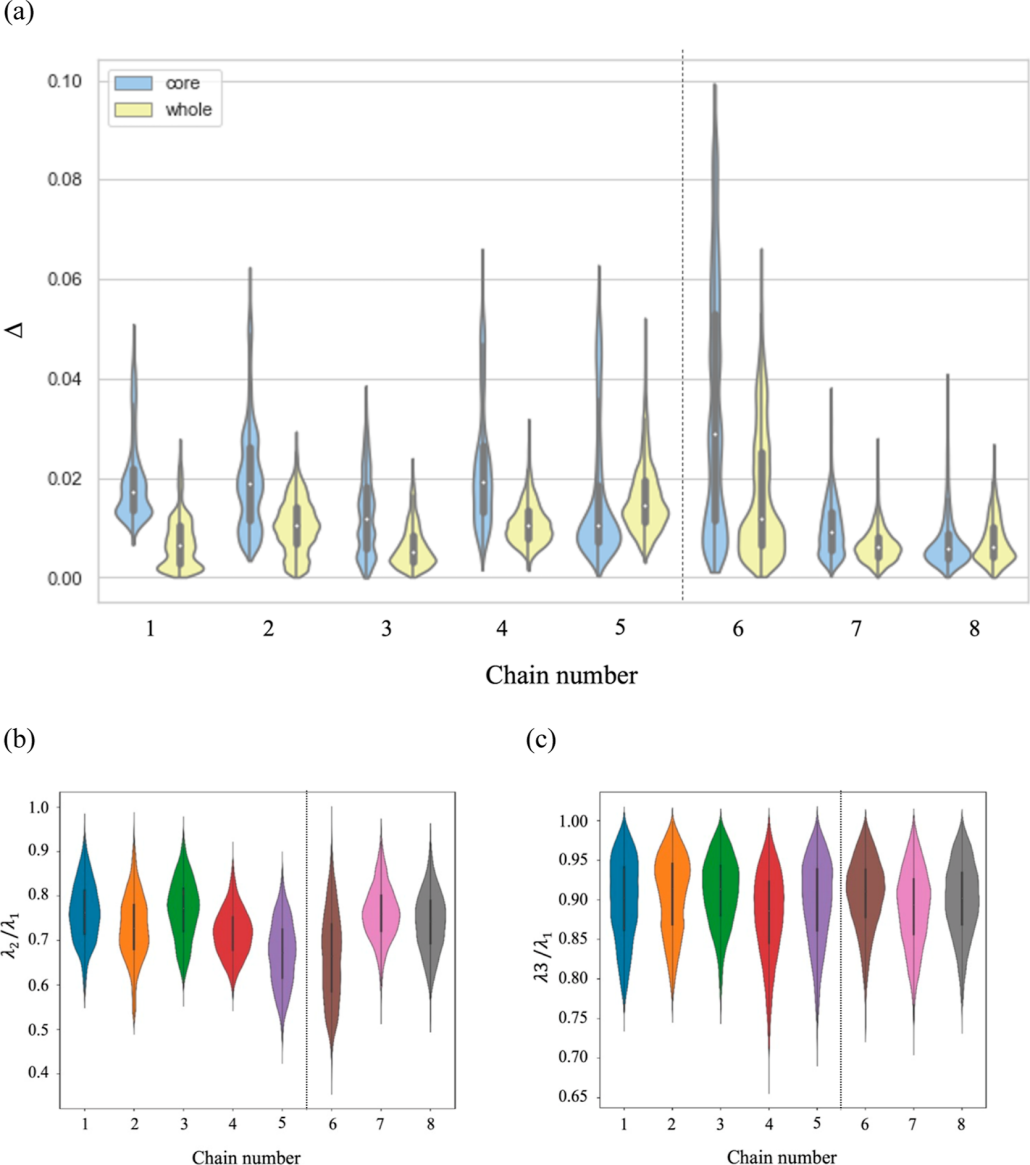
(a–c) (a) Violin plots depicting the asphericity for the core of the micelle (light yellow) and the entire micelle (light blue). (b) Violin plots depicting the ratios of the eigenvalues λ_2_/λ_1_ obtained from the inertial gyration tensor. (c) Violin plots depicting the ratios of the eigenvalues λ_3_/λ_1_ obtained from the inertial gyration tensor. Note that the kernel density estimation (KDE) was used to smoothen the data in the representative violin plots.^79,80^

The asphericity value of the polypeptoid micelles was calculated for that of the entire micelle and that of the hydrophobic core of the simulated micelle (Figure 7a). Figure 7b,c shows the ratios of λ_2_/λ_1_ and λ_3_/λ_1_, respectively, obtained from the gyration tensor of the micelles formed by chains 1 to 8. If the value of these ratios is close to 1, the shape of the micelle is more spherical. Otherwise, the micelle is elongated. The aspherical nature of micelles can be roughly qualified and confirmed by inspecting the snapshots from the simulation trajectories. In general, the asphericity for the entire micellar structures formed by the singly charged series displayed a slightly increasing trend from the micelles formed by chain 1 to that of chain 5, while the opposite was observed for the triply charged series (Figure 7a). Overall, the asphericity parameter of the micelles formed by chains 1–4 in the singly charged series and chains 7 and 8 in the triply charged series were generally spherical with a calculated asphericity of approximately 0.01 or less (Figure 7a), while chains 5 and 6 are atypical. Unsurprisingly, the micellar structure formed from chain 5 is the least spherical in the singly charged series, given the proximity of the charged group to the hydrophobic core, and compared to the micelles formed by chains 1–4 was also observed to have the broadest distribution in the asphericity of the entire micellar structure. In general, as one goes from chain 1 to 5 in the singly charged series, the asphericity increases, and this can be attributed to the interplay between the effective electrostatic repulsion among the charged monomer (through ionic monomer position from the hydrophobic block), the propensity of hydrophobic blocks to minimize interaction with water (i.e., hydrophobic effect), and solvation of the ionized monomers via ionic–dipolar interaction. However, for the micelles formed by the triply charged series, the violin plots of the asphericity parameter illustrated that the ionic monomer position was not the sole determinate of the resulting asphericity of the micellar structures (Figure 7a). If this were the case, it would be expected that the micelle formed by chain 6 (with the charged monomers furthest from the hydrophobic segment) would be the least aspherical, while the micelle formed by chain 8 would be the most aspherical. However, the micelle formed by chain 6 (oligomer with the charged groups furthest from the hydrophobic segment) demonstrated the most significant deviation from an ideal sphere (Figure 7a). Additionally, the plots of the ratios of the eigenvalues of the *x*, *y*, and *z* components demonstrate that micellar conformations formed by chain 6 have a much smaller λ_2_ than λ_1_, which suggests that one side was extended and, therefore, contributed to the deviation from an ideal spherical conformation (Figure 7b,c). For the micelles formed by chains 1–4, 6, and 7, the core of the micelle generally was more aspherical than when the asphericity was calculated for the entire micelle. Interestingly, the asphericity of the micelles formed by chains 5 and 8 did not exhibit much difference between the core and the whole micelle (Figure 7a). Although their aggregation numbers, position of the ionic monomer(s), and *R*_g_ were similar, the micelle formed by chain 5 was much more nonspherical than that formed by chain 8. This indicated that the asphericity is not necessarily correlated to the monomer position or *R*_g_ (size) of the micelle.

### Shape

**Figure 8:**
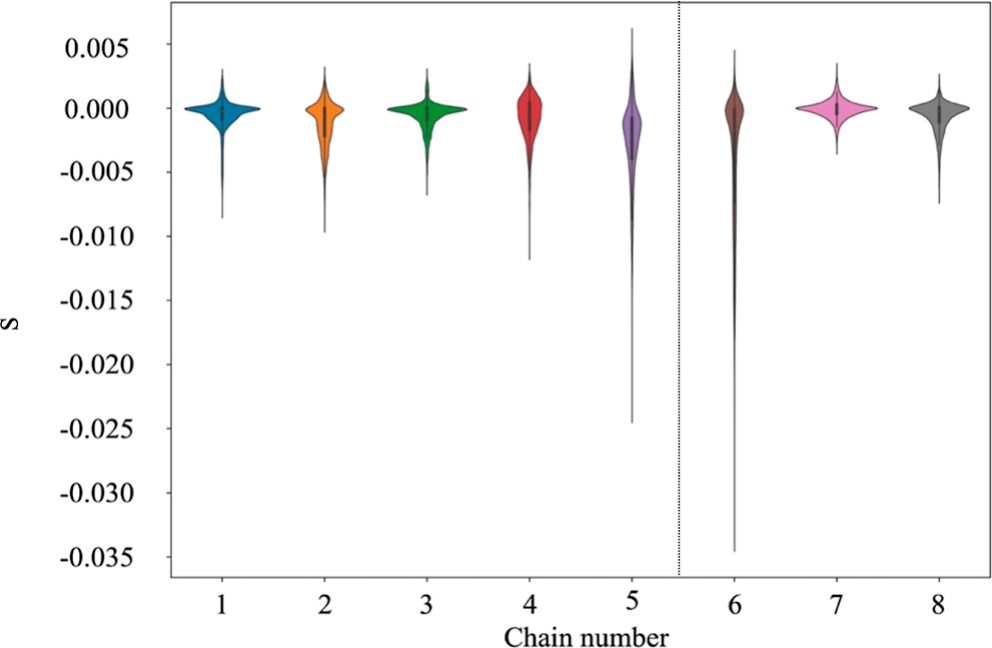
Violin plots of the overall shape for the entire micelle for chains 1 to 8. Note that the KDE was used to smoothen the data for the representative violin plot.^79,80^

The shape parameter for the formed micelles revealed that the micelles formed by chains 1–8 have a shape parameter ranging from approximately −0.035 to 0.005 (Figure 8). In general, the shape parameter for the micelles formed by chains 5 and 6 were slightly more oblate than the micelles formed from chains 1–4 and chains 7–8 that had a shape parameter closer to zero (Figure 8). While the shape parameter of the micelles tended toward slightly negative values (indicating slightly oblate shapes), further examination of the violin plots depicting the distribution in shape parameter showed that the shape parameter also extends into the positive values of shape (*S* > 0), indicating the possibility for a small distribution of prolate shapes as well (Figure 8). Compared to the micelles formed by chain 1–4 and chain 7–8, the micelles formed by chains 5 and 6 have much broader distributions in the shape parameter, ranging from slightly prolate to a much deeper extension into the oblate regions. From inspecting the MD trajectory of especially chain 6 compared to the micellar systems, it was evident that there were more fluctuations in the micelle’s shape with a clear oscillation between prolate and oblate structures.

### Solvent-Accessible Surface Area

**Figure 9:**
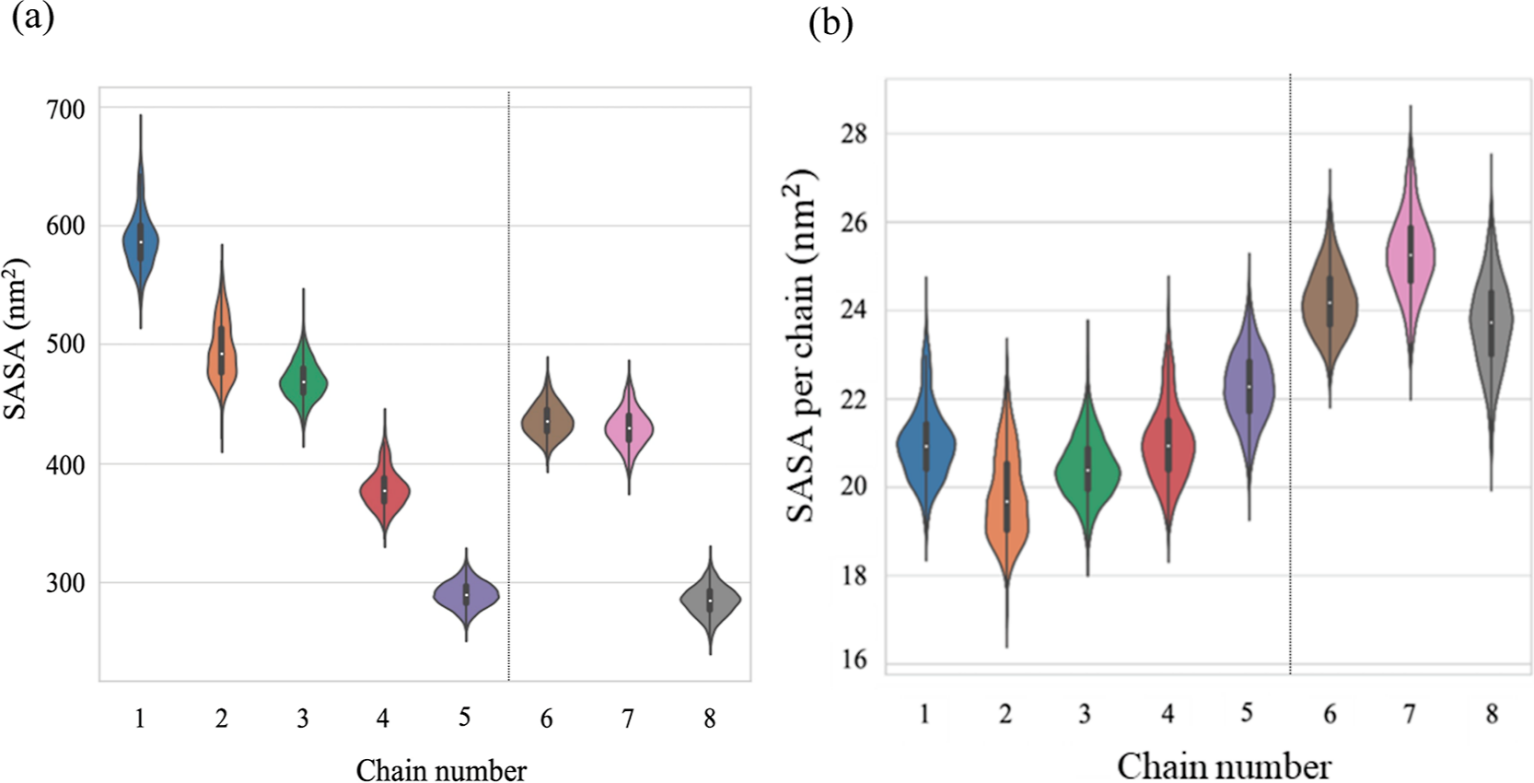
Violin plots of (a) total SASA of the micelles and (b) SASA per chain in micelle for those formed by chains 1–8. Note that the KDE was used to smoothen the data for the representative violin plots.^79,80^

The average total SASA of each micelle and the SASA per chain were calculated and tabulated in Table 2 to assess the differences in the 3D structure of micelles as a function of the number of charged moieties and their positions along the polypeptoid chain. Violin plots of the total SASA and the SASA per chain were also constructed to visualize the distributions in these two parameters (Figure 9).

**Table 2:** Average Total SASA of the Micelles, SASA per Chain, the Ratio of Total SASA to the Indicated Reference, and the Ratio of the Radius of Gyration Squared (*R*_g_^2^) to the Indicated Reference (Chain 1)

chain no.	SASA (nm^2^)	SASA/chain (nm^2^)	SASA/SASA_micelle1_	*R*_g_^2^/*R*_g(micelle1)_^2^
1	588 ± 4	21	1:1 (reference)	1:1 (reference)
2	495 ± 5	20	1.19:1	1.11:1
3	469 ± 3	20	1.25:1	1.18:1
4	378 ± 2	21	1.56:1	1.37:1
5	289 ± 3	22	2.03:1	1.59:1
6	437 ± 4	24	1.35:1	1.10:1
7	429 ± 4	25	1.37:1	1.14:1
8	284 ± 2	24	2.07:1	1.67:1

The calculated SASA per individual surfactant chain was determined by dividing the total SASA by the aggregation number of each simulated micelle to quantitatively understand the compactness of each micellar structure (Table 2, Figure 9b). Based on the calculated SASA per chain, it was found that SASA per chain for the singly charged series was relatively the same, ranging between values of 20–22 nm^2^. The calculated SASA per chain within the triply charged series (chain 6–8) was relatively the same but larger than the calculated SASA for the singly charged series ranging between 24 and 25 nm^2^. Larger values for SASA per chain observed for the triply charged series indicated that, on average, each chain in the micellar structures formed by the triply charged series had more access to the surrounding solvent and suggested that these micellar structures were less compact than those for the singly charged series. In addition, it was interesting to observe that the calculated SASA per chain for the micellar structure formed by chain 5 was slightly larger than its singly charged counterparts, indicating that the structure of the micelle formed from chain 5 was less compact than the micelles formed by the rest of the singly charged series.

Not surprisingly, the total SASA value was proportional to the micelle size. Therefore, it was reasonable to expect that the surface of the micellar structure formed by chain 1 was the most exposed to the surrounding water, while the surface of the micelles formed by chains 5 and 8 was the least exposed to the surrounding water. Comparing the *R*_g_ and the total SASA of each micelle’s structure can give insights into the roughness of each micelle’s surface and additional insights into the shape of the micelle. Assuming a perfectly spherical symmetry, the ratio of the average total SASA for the micelle formed by two different chains should be approximately equal to the ratio of *R*_g_^2^ of each micelle because the surface area of an ideal sphere with the hydrodynamic radius *R*_H_ (which is 4π*R*_H_^2^) is directly proportional to *R*_g_^2^ (since *R*_g_ = 0.775*R*_H_ for a perfect sphere). Owing to the rough nature of the micelle surfaces, the average SASA value of each micelle was larger than that of a perfect sphere of the same size. For each micelle, chain 1 was used as a reference to calculate the ratios of the total SASA and ratios of *R*_g_^2^ with the micelles formed by chains 2–8 (Table 2). Although there is surface roughness, there is an overall average spherical symmetry for these micelles because the calculated SASA ratios in comparison to their corresponding ratios of *R*_g_^2^ are approximately equal for all the formed micelles (Table 2). As the ionic monomer is moved toward the core of the micelle, the difference between the calculated ratio of the average total SASA and their corresponding ratios of *R*_g_^2^ for the micelles formed by chain 1 in comparison to chain 2–5 resulted in a greater difference between the ratio of average total SASA between the two micelles and the ratio of *R*_g_^2^ of each micelle (except for chain 2). Interestingly, this trend is similar to the trend in the asphericity of the singly charged series. MD simulation trajectories clearly show that although the charged groups in chain 5 are close to the hydrophobic groups, they are nonetheless exposed to the solvent. The slightly larger SASA per chain for chain 5 compared to the rest of the singly charged series, coupled with the smaller aggregation number indicates that the hydrophobic core is not perfectly screened by the hydrophilic section, and the micelle formed by chain 5 is less compact. Combining this result with the comparatively aspherical nature of the micelle suggests that the micelle formed by chain 5 is elongated since the charged groups want to remain as solvated as possible. Although one could expect a similar trend for the triply charged series as the charged groups are moved toward the micelle’s core, the calculated SASA ratios in comparison to their corresponding ratios of *R*_g_^2^ were not similar to the trend in the asphericity of the triply charged series, suggesting the importance of noncovalent interactions other than charge (COO^–^)–dipole (water) interactions in dictating the shape for the triply charged case.

### Comparative Analysis of Shape Descriptors

2D joint plots with kernel density estimation can expose bivariant relationships in addition to the distributions of the individual attributes. The 2D joint plot of SASA versus *R*_g_ of the sequence-defined polypeptoids generally had one maximum except for the micellar structure formed by chain 6 (Figure S7a). The single maxima observed for the micellar structures formed by chains 4, 5, and 8 were more intense compared to the single maxima formed by chains 1, 2, 3, and 7. This difference indicated that while there was one dominant micellar structure formed by chains 1, 2, 3, and 7, these micellar structures were more diffuse compared to those formed by chains 4, 5, and 8.

**Figure 10:**
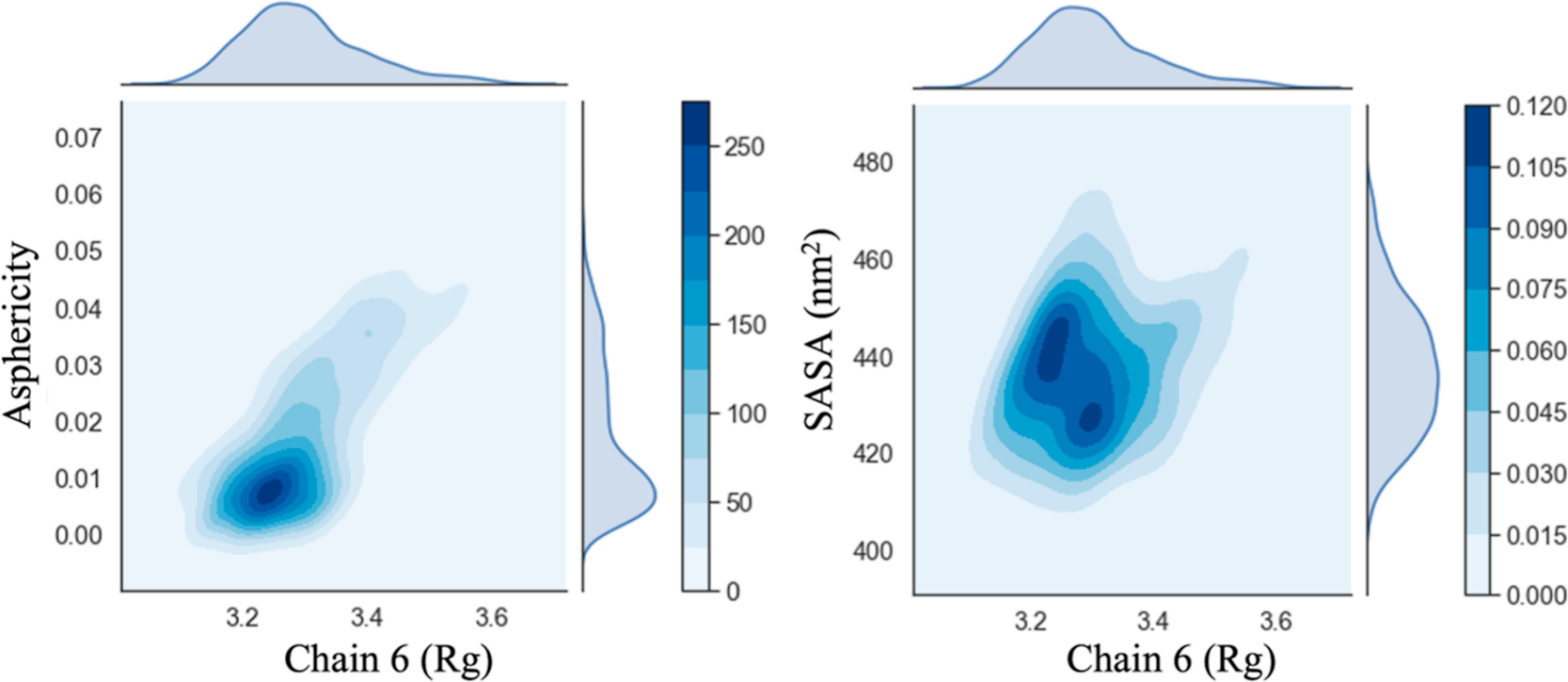
2D joint plots of *R*_g_ (nm) versus asphericity and *R*_g_ (nm) versus SASA (nm^2^) for chain 6. Note that the KDE was used to smoothen the data for the representative 2D joint plots.^79,80^

On the other hand, the distribution for chain 6 (see Figure 10) had two distinct maxima indicating the possibility of two distinct conformations with a similar *R*_g_ or size. Although these two maxima observed in the plot of SASA versus *R*_g_ are similar in the spread in *R*_g_, the SASA distribution can extend between 425 and 440 nm^2^. Moreover, the 2D joint plots of SASA versus asphericity showed that the micellar structures formed by chains 5 and 6 have a narrower distribution in SASA but have broader asphericity distributions than the other systems (Figure S7b). The micellar structures formed by chains 3, 4, 7, and 8 also showed a single, dominant conformation in the 2D joint plot of SASA versus asphericity (Figure S7b). The information obtained from the 2D joint plot of SASA versus *R*_g_ combined with the plot of SASA versus asphericity suggested a single spherical dominant structure arises for the case of chains 3, 4, 7, and 8. However, the micellar structure formed by chain 5 appeared to be more aspherical and with a relatively wider distribution in the asphericity and *R*_g_ but with a comparatively narrower distribution in SASA, suggesting that this micelle is more diffuse than the micelles formed by chains 3, 4, 7, and 8. The micelle formed by chain 6, on the other hand, shows a wide distribution in the plot of SASA versus asphericity and SASA versus *R*_g_, indicating that its structure can vary from spherical to elongated conformations, which was also evident from inspecting the MD trajectory of the micelle formed by chain 6 (Figure 10). It is also worth mentioning that chains 1 and 2 have a diffuse distribution of conformations compared to those formed from chains 3, 4, 7, and 8. The 2D joint plots of asphericity versus SASA further supported the observation that the micellar structures formed by chains 3, 4, 7, and 8 were relatively spherical, while chain 6 moves between spherical to oblate conformations (Figures S7b and 10). Even though the micelles formed by chain 1 appeared more diffuse in structure, the resulting structures were primarily spherical.

In addition to the 2D distributions, the Pearson correlation coefficients between the *R*_g_ and the asphericity, shape, and SASA were determined using the SciPy library for the micelles formed by chains 1–8 and are tabulated in Table S2. Correlation coefficients measure the degree of the linear relationship between two attributes, in this case, *R*_g_ and the other attributes of the micellar structures. In general, there is generally a positive correlation between *R*_g_ and the SASA of the micelle, which is excepted. As previously mentioned, assuming a perfect spherical symmetry, the ratio of the average total SASA for the micelle formed by two different chains should be approximately equal to the ratio of *R*_g_^2^ of each micelle since the surface area of an ideal sphere with the hydrodynamic radius *R*_H_ (which is 4π*R*_H_^2^) is directly proportional to *R*_g_^2^ (since *R*_g_ = 0.775*R*_H_ for a perfect sphere). However, the correlation is not as strong with chain 6, which is in keeping with the 2D distribution (Figure S7a) that suggests that there are two different stable conformers. *R*_g_ and the asphericity show a moderate positive correlation for the singly charged chains, with the asphericity, on average, increasing with an increasing *R*_g_. However, for the triply charged case, there is a strong positive correlation between the two for chain 6, whereas for chains 7 and 8, it is negligible. The correlation between *R*_g_ and the shape of the micellar structure for almost all the chains is very slightly negative. However, for chains 2 and 6, the correlation is stronger (negative correlation), with the micelles becoming more oblate with an increasing *R*_g_ (see 2D plot Figure S7d). Chain 7 shows a very little change from an essentially spherical shape with an increasing *R*_g_ (2D distribution Figure S7d), and hence, the correlation is negligible. This analysis further suggests that *R*_g_ by itself is an insufficient descriptor of the change in the micellar shape and structure with a change in the charge position and number of charges.

### Analysis of Charge–Sodium Interactions

**Figure 11:**
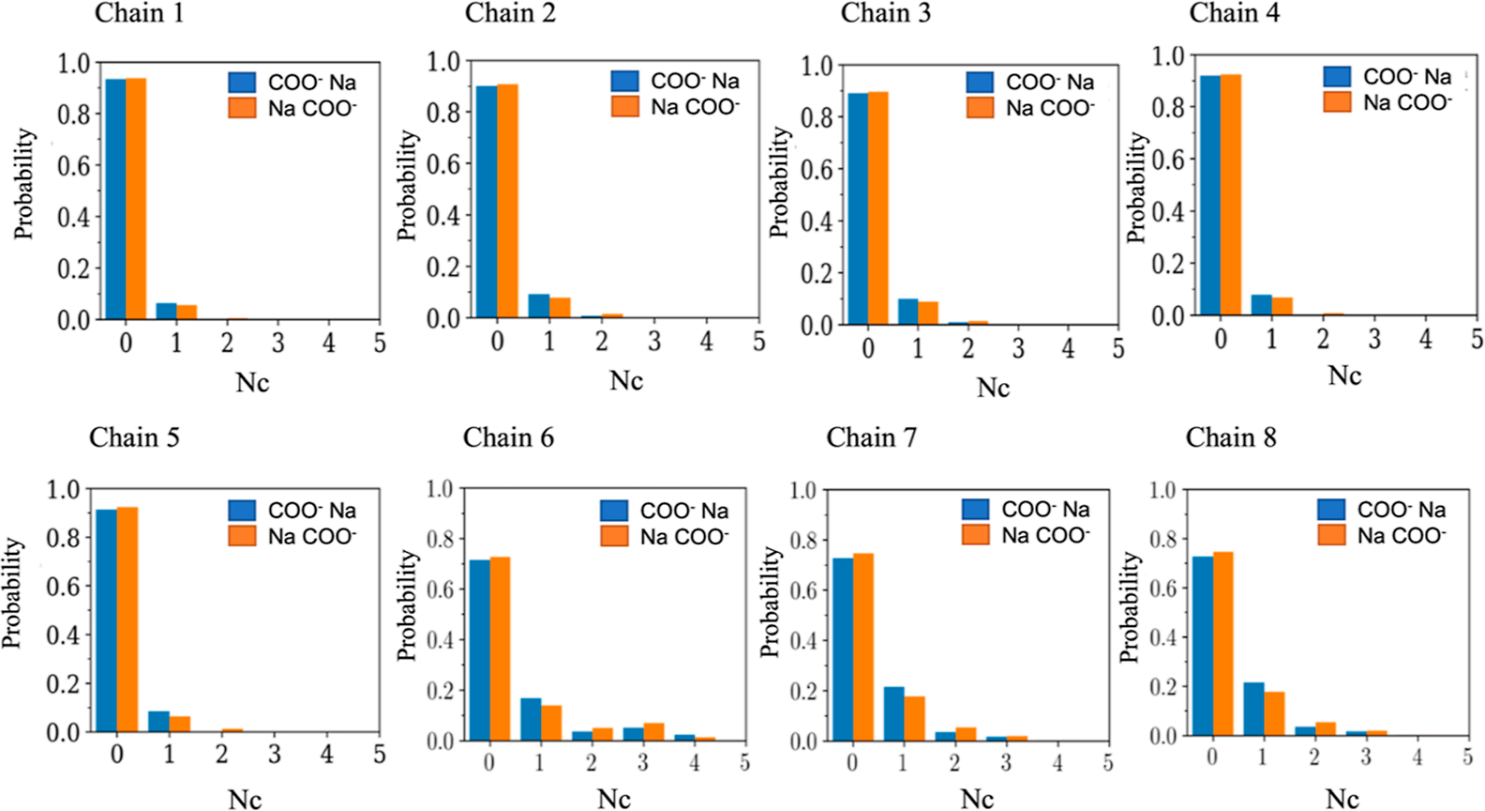
Distribution of the number (*N*_C_) coordinated sodium atoms around the carboxylate groups (COO^–^) to sodium ion (blue) and the number of sodium atoms around COO^–^ (orange) within 0.35 nm.

The effect of noncovalent interactions, such as electrostatic repulsion, charge–sodium association, charge neutralization, and charge–dipole interactions, on dictating the structure of each micelle was investigated. The distribution of the number of the coordinated sodium ions around the carboxylate groups (using the oxygen atoms of COO^–^) was plotted to evaluate the contribution of the charge–sodium association for each micelle formed by chains 1–8 (Figure 11). A cutoff distance of 0.35 nm between the Na^+^ ion and the oxygen atom of the carboxylate group was used to define a sodium ion as coordinated to the charged group of the micelle, as this is the first minimum in the Na^+^–oxygen (COO^–^) radial distribution function (Figure S8). It is clear that almost 90% of the sodium counterions were not condensed for the micelles formed by the singly charged micelles, while approximately 70% of the sodium counterions were not condensed for the micelles formed from the triply charged series.

This observation indicated that the charged carboxylate group preferred to be hydrated by the surrounding water molecules rather than associate with a sodium counterion for both the singly charged series and the triply charged series. However, the charged carboxylate group(s) in micelles formed by the triply charged series were 20% more likely to ion-pair with a sodium ion than the carboxylate group in the micelles formed by the singly charged series, suggesting a greater sodium affinity for the charged carboxylate group in the micelles formed by the triply charged series than the singly charged series.

### Analysis of the Charge–Charge Repulsion and Salt-Bridge Effect

**Figure 12:**
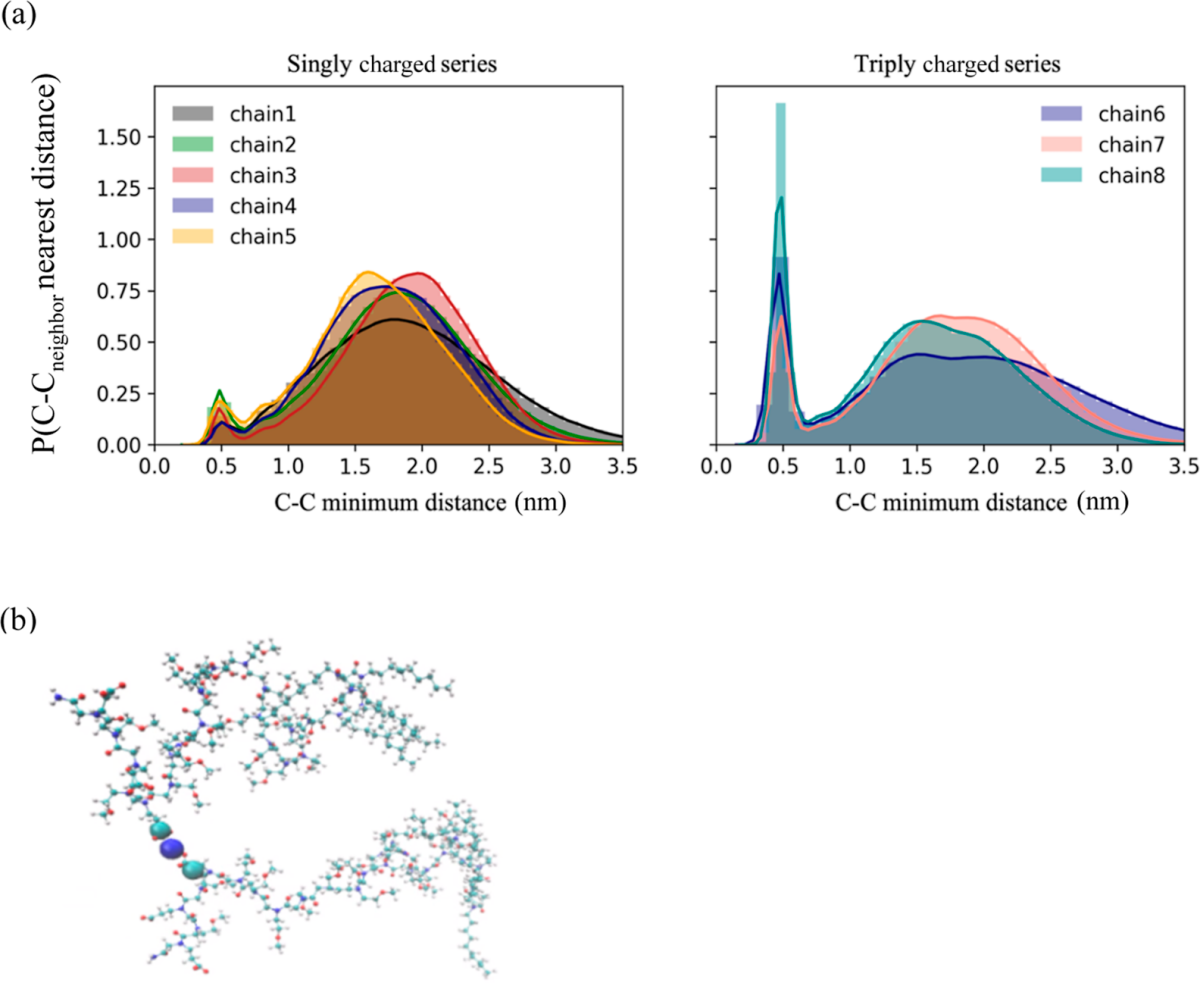
(a) Probability density function (*P*) of carboxylate carbon (C)–carboxylate carbon (C) closest neighbor distance (nm) located on a different polypeptoid chain within the micelle. (b) Snapshot illustrating the sodium ion (blue sphere) bridging two COO^–^ groups from different peptoid polymer chains in the micelle. The histograms can be seen in light transparency. Note that the KDE was used to smoothen the data, and the unsmoothened data is shown as well in the graphs.^79,80^ All distributions were normalized to have a unit area.

The closest carbon atom of a carboxylate located on a different polypeptoid chain within the micelle was determined for every carbon atom of a carboxylate. The distribution of this carboxylate carbon (C)–carboxylate carbon (C) closest neighbor distance (nm) (C–C_neighbor_ nearest distance) was used to evaluate the contribution of electrostatic repulsion for each micellar system (Figure 12a). For the micelles formed by the singly charged series (chains 1–5), the distributions of C–C_neighbor_ nearest distance were similar within this series consisting of a broad peak with a maximum of 1.5 to 2.0 nm, in addition to a significantly smaller peak centered around 0.5 nm. The distribution of the C–C_neighbor_ nearest distance for the micelles formed by the triply charged series also depicted a similar broad peak with the maximum within similar ranges as observed for the micelles formed by the singly charged series (Figure 12a). This predominant peak that was consistent across all the studied micellar structures indicates that the carboxylate groups from different polypeptoid chains within a micelle attempt to optimally orient themselves within a specific distance from one another to minimize the extent of electrostatic repulsion, providing support for the hypothesis that electrostatic repulsion is the primary determinant of the experimentally determined aggregation number for each micellar system.

Since it was expected that the charged carboxylate groups would be oriented to minimize the extent of electrostatic repulsion, it was unusual to observe a significant fraction of carboxylate group(s) from different polypeptoid chains coming within 0.5 nm of one another for the micelles formed by the triply charged series as compared to the singly charged series. More significant electrostatic repulsion was expected from the micelles formed from the triply charged series due to an increased charge content with respect to the singly charged series. Therefore, an intense, narrow pre-peak around 0.5 nm warranted a more comprehensive analysis of the sodium–carboxylate interactions (Figure 12a). Qualitative analysis of the MD trajectories illustrated that it was possible for the carboxylate groups of separate polypeptoid chains to come relatively close to one another when a sodium ion was located directly between two carboxylate anions of different polypeptoid chains within the micelle forming a COO–Na–COO “salt bridge,” as illustrated by the snapshot in Figure 12b. It was clear from Figure 12a that for the micelles formed by the triply charged series, there were a larger fraction of negatively charged chains that are much closer (a pre-peak at around 0.5 nm) as compared to the micelles formed by the singly charged series.

The correlation between the C–C_neighbor_ nearest distance within a defined distance and the extent of the COO–Na–COO salt bridge effect for these carboxylate groups was investigated in an attempt to explain this pre-peak at around 0.5 nm in Figure 12a. The analysis was performed by first calculating the distance between the closest carbon atom of a carboxylate located on a different polypeptoid chain within the micelle and subsequently between all sodium ions and those selected nearest two carboxylate carbons on different chains. A sodium ion was considered bridging between the two carboxylates of separate polypeptoid chains if the sodium was within a cutoff distance of 0.5 nm of both carboxylate groups. In the analysis of the salt bridge fractions, the maximum C–C_neighbor_ nearest distance was defined for two distinct cases: when the C–C_neighbor_ nearest distance was less than or equal to 0.5 nm and when the C–C_neighbor_ nearest distance was less than or equal to 1.0 nm. The results in Table 3 showed that when the C–C_neighbor_ nearest distance was defined to be less than or equal to 0.5 nm, up to 99% or more of those associations occurred as salt bridges for the triply charged series. In contrast, for the singly charged series, the fraction of salt bridges when the C–C_neighbor_ nearest distance between chains was defined to be less than or equal to 0.5 nm was slightly lower than 99% and ranged between 86.3 and 98.3%. At the same time, the proportion of salt bridges when the C–C_neighbor_ nearest distance was defined to be less than or equal to 1.0 nm was significantly lower for both the micelles formed by the singly charged and triple charged series. For the singly charged series, the results in Table 3 revealed that the fraction of salt bridges for a defined C–C_neighbor_ nearest distance of 1.0 nm or less ranged between 15.7 and 36.2%, while the fraction of salt bridges was between 70.4 and 75.6% for the micelles formed by the triply charged series. It was evident from the combined results of Figure 12a and Table 3 that a shorter C–C_neighbor_ nearest distance (especially under 0.5 nm) was strongly correlated with more carboxylate-sodium–carboxylate salt bridges.

**Table 3:** Salt Bridge Fractions When the Distance of the Carbon Carboxylate to the Nearest Neighboring Carbon Carboxylate on Another Polypeptoid Chain (C–C_neighbor_ Nearest Distance) is Closer Than 0.5 and 1.0 nm, Respectively

chain no.	C–C_neighbor_ < 0.5 nm (%)	C–C_neighbor_ < 1.0 nm (%)
1	86.8	15.7
2	95.9	36.2
3	98.3	35.6
4	91.0	22.4
5	88.9	32.3
6	99.7	74.4
7	99.4	70.4
8	99.8	75.6

It was initially expected that electrostatic repulsion was the sole driving force of the resulting aggregation numbers and the *R*_g_ for the singly charged series and the triply charged series, but charge–charge repulsion is not the only interaction determining the shape and asphericity of each micelle as previously mentioned. Prominent carboxylate–sodium–carboxylate salt bridges between polypeptoid chains combined with higher sodium counterion associations in the triply charged series compared to the singly charged series suggested that carboxylate–sodium interactions compete with electrostatic repulsion to modulate the shape and the asphericity of the micelle. For systems with similar aggregation numbers in the micelles formed by chains 4 (*N* = 18), 6 (*N* = 18), and 7 (*N* = 17) and the micelles formed by chains 5 (*N* = 13) and 8 (*N* = 12), an explanation for these significant differences between the singly charged series and the triply charged series implies that the aggregation number and *R*_g_ are incomplete descriptors of determining the shape and structure of these micelles.

### Analysis of Charge–Water (Solvent) Interactions

**Figure 13:**
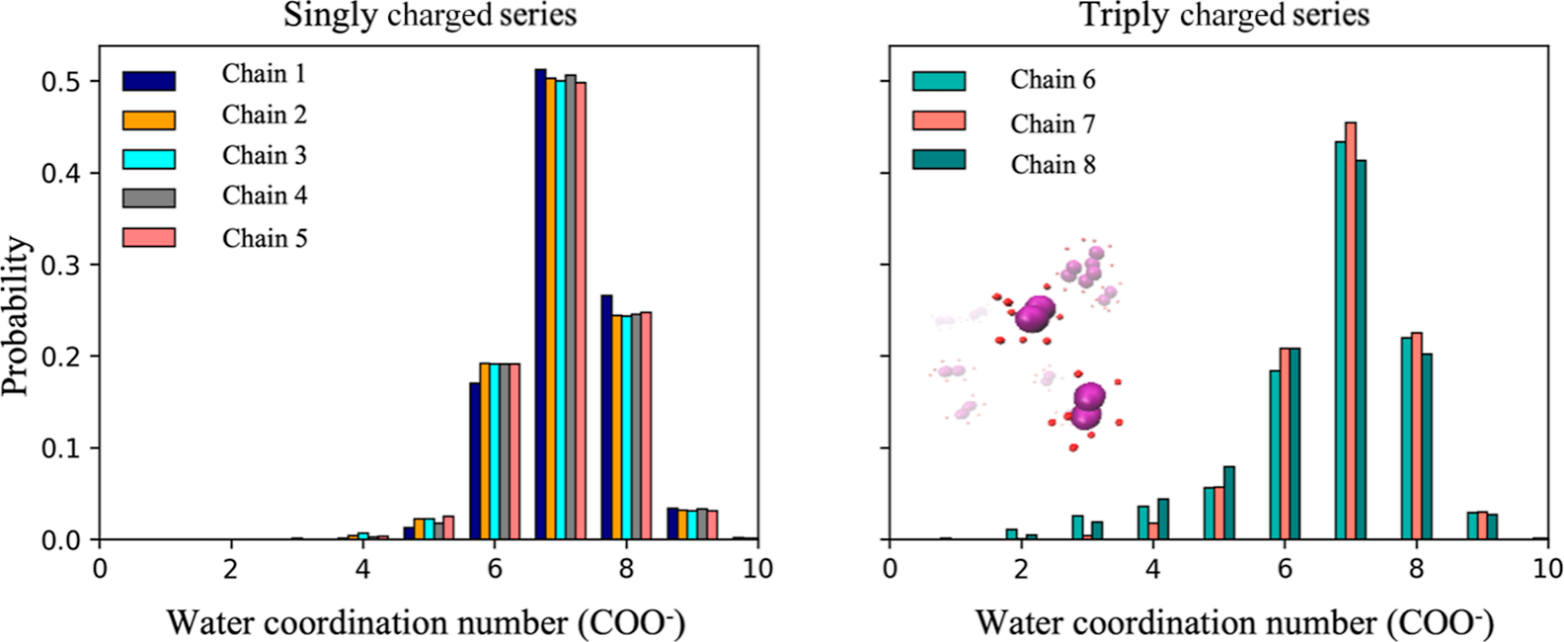
Probability distribution of water coordination to the COO^–^ charged group of block copolymer micelles for the singly charged (left) and triply charged (right) series. (Inset-right) A snapshot of water molecules (O atoms only shown in red) coordinated to the COO^–^ (O atoms only shown in purple) charged group of block copolymer micelles for chain 6.

The distribution of the number of water molecules coordinated with the COO^–^ group was plotted to understand the contribution of charge (carboxylate group)–dipole (water/solvent) interactions to the micellar structure (Figure 13). A cutoff distance of 0.35 nm between the oxygen atom of water and the oxygen of the carboxylate group was used to define as coordinated to the charged group of the micelle (Figure S9). This distribution for the micelles formed by the singly and triply charged showed that seven water molecules tend to solvate each carboxylate ion (Figure 13). When the position of the carboxylate ion was closer to the hydrophobic segment (chains 5 and 8), the COO^–^ ion was solvated almost to the same extent as when it was the furthest from the hydrophobic segment (chains 1 and 6). This observation suggested that the ionic group prefers to be solvated even when close to the hydrophobic core, enabling the COO^–^ groups to be close to each other by partially screening the negative charge. For the singly charged series, the highest probability of the coordination number was seven water molecules, followed by eight and six coordinated water molecules. The COO^–^ groups from the micelles formed by the singly charged series also had a higher probability of water coordination than the COO^–^ groups from the triply charged series, for there were significant fractions of COO^–^ groups in the triply charged case with only three, four, and five water molecules. Both micelles formed from chains 6 and 7 have three charged groups per chain, and although they share very similar aggregation numbers and sizes, the three charged groups in chain 6 are further away from the hydrophilic/hydrophobic junction of each polymer chain and, therefore, were expected to be more exposed to surrounding water molecules. Combined with the results from Figures 11 and 12a that showed the COO^–^ groups from the micelles in the triply charged series were also more likely to associate with sodium ions, it was unsurprising that the probability of the COO^–^ group coordinating to a more significant number of water molecules decreased. This observation provides an explanation to why the micelles formed by the triply charged series (especially chain 6) have lower water coordination numbers.

## Conclusions

In this work, the all-atom simulations provide a detailed understanding of micelles’ structural aspects formed by sequence-defined peptoid block copolymers. Our results revealed the importance of solvent (dipole)–ionic group (charge) interactions in addition to charge–charge interactions on the stability and structure of self-assembled charged polypeptoids as a function of the placement of the charged group on the backbone. We further elaborated on the atomistic structural properties of the micelles formed in aqueous solution by probing the size, SASA, asphericity, and shape of the micellular structure, as well as the contributions of charge solvation, sodium–carboxylate interactions, and electrostatic repulsion to these various structural properties. In this study, we demonstrated the importance of charge (COO^–^ group)–water solvent (charge–dipole) interactions on the shape of the micelles formed by the singly charged series. As the charged monomer is moved toward the hydrophilic/hydrophobic junction, the micelle will deviate from an ideal spherical micelle to ensure all charges are solvated. On the other hand, the shape of the micelles formed by the triply charged series was further complicated by greater charge–sodium interactions which compete with charge–charge repulsion and charge–water (dipole) interactions, making the shape of the micelle more challenging to predict and, therefore, less tailorable. The calculated asphericity parameter illustrated that the ionic monomer position was not the sole determinate of the resulting asphericity of the micellar structure of the micelles formed from the triply charged series. If this were the case, it would be expected that the micelle formed by chain 6 (with the charged monomers further from the hydrophobic segment) would be the least aspherical, while the micelle formed by chain 8 would be the most aspherical. However, the asphericity of the micelle formed by chain 6 does not follow that trend. Combining the observation that chain 6 has a higher probability of a higher sodium coordination (*N*_c_ > 3) than the micelles formed by chains 7 and 8 with the fact that the ionic monomers on chain 6 are positioned furthest away from the hydrophobic block in comparison to those on chain 7 or chain 8, allows for the potential flexibility of this micelle to maximize neutralization through salt bridge formation. In the attempt to find a conformation in which these salt bridge interactions are maximized with surrounding sodium atoms, the micelle formed by chain 6 will sample various configurations contributing to the wide distribution in the asphericity of the entire micelle and the significantly larger distribution in asphericity of the core of the micelle. It can be reasoned that when the position of the charged monomers is closer to the hydrophobic block, as in chains 7 and 8, it is difficult for the micelle to rearrange to different conformations, therefore resulting in generally more spherical structures with unimodal distributions in asphericity. In comparison to the micelles formed by the singly charged series, the micellar structures formed by the triply charged series, in general, have a greater affinity for sodium than the singly charged series resulting from a greater charge density from just increasing the number of charged monomers along the polypeptoid chain. More significant charge density not only results in a greater effective electrostatic repulsion but also a need to accommodate greater effective electrostatic repulsion through salt bridge interactions. Because the formation of these salt bridge interactions is essential to maintaining the micellar structure of the micelles formed by chains 7 and 8, especially when the effective electrostatic repulsion is expected to be greater as the ionic monomers are closer to the hydrophobic block of the polypeptoid chain, it is possible that the micelle formed by chains 7 and 8 do not have the same flexibility to rearrange or deform once the hydrophobic core collapses to minimize interaction with the surrounding water molecules. As a result, the micelle formed by chain 8 is constrained to the dominant conformation in which the hydrophobic core of the micelle minimizes interaction with the water while maximizing favorable salt bridge interactions. Understanding the relationship between the electrostatic sequence and subtleties in the aggregation nanostructure (shape) has various applications in drug delivery and designing phase behavior materials (i.e., complex coacervates). In future work, we will investigate the effect of changing the pH and salt type on the micelle structure and study the effect of mixing different sequence-defined peptoid oligomers as employed in this study.

## Supporting Information Available

Additional topology information, force field information, time evolution of *R*_g_, probability density distributions of asphericity, additional information on violin plots, additional data for *R*_g_, supplemental 2D joint plots, Pearson correlation coefficients, and plotted radial distribution functions (PDF)
