# Supplementary material for: Unraveling the Role of Charge Patterning in the Micellar Structure of Sequence-Defined Amphiphilic Peptoid Oligomers by Molecular Dynamics Simulations
Source: Macromolecules. 2022 Jun 14;55(12):5197–212. doi: 10.1021/acs.macromol.2c00141 (PMC9245439; doi:10.1021/acs.macromol.2c00141)
Supplement: Supplementary file 1 — ma2c00141_si_001.pdf [file ma2c00141_si_001.pdf]

Unraveling the Role of Charge Patterning in the Micellar Structure of Sequence Defined Amphiphilic Peptoid Oligomers by Molecular Dynamics Simulations

Erin Tsai,[1†] Hishara Keshani Gallage Dona,[1†] Xinjie Tong,[1†] Pu Du,[1] Rolf David,[1] Brian Novak,[2] Steven W. Rick,[3] Donghui Zhang,[1] and Revati Kumar[1,4 *]

[1]Department of Chemistry, Louisiana State University, Baton Rouge, Louisiana 70803, United States

[2]Department of Mechanical and Industrial Engineering, Louisiana State University, Baton Rouge, LA 70803, United States

[3]Department of Chemistry, University of New Orleans, New Orleans, Louisiana 70148, United States

[4]Center for Computation and Technology, Louisiana State University, Baton Rouge, LA 70803, United States

Topology and Force Field Information

To create the topology for different polypeptoid block copolymers more efficiently, we constructed three regular residues (DEC, MOE and COE) and their corresponding terminated residues (HPT, TMT and TPT) first. The .mol2 files and topology with forcefield parameters used in this work can be found on the following GitHub website: https://github.com/pandatt66/Peptoid. The initial structure of the polypeptoid can then be easily assembled depending on the desired length of the copolymer and position of the charge. We then used LEaP[59] program to build copolymers and generate Gromacs compatible topology via ACPYPE[59]. Examples of commands can be found in the README file in the same GitHub repository.

(a).

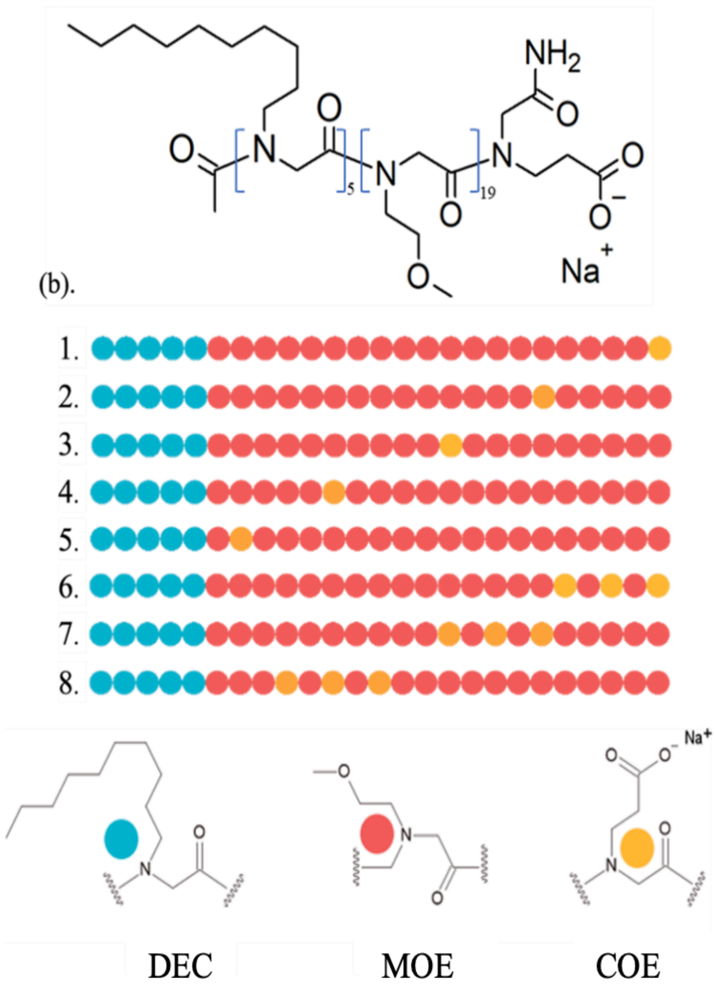

Figure S1. (a) Chemical structure represents singly charged series of ionic peptoid block copolymers, (b) the sequence of the studied singly and triply charge peptoid identified from chain 1 to 8, which are composed of three basic repeated units.

Choice of Simulation Time Length

The time evolution of the radius of gyration (Rg) per configuration of the micelles formed by chains 1-8 was plotted over the simulation time of 100 ns to check for simulation convergence from each of the six initial starting configurations (Figure S2). It was observed that after the initial 50 ns of simulation time from the six initial starting configurations for the representative micellar systems, the Rg (nm) over the simulation time (ns) remains relatively steady indicating convergence (Figure S2). Furthermore, the comparison of the generated distributions from the probability density function P(asphericity) of the representative chains 1-8 illustrated that although similar in asphericity, the generated distributions of the probability density function from the last 50 ns of total 100 ns of simulation time for each configuration are not necessarily identical (Figure S3) indicating that the starting structures are not necessarily identical and exhibit differences.

Time Evolution of Radius of Gyration

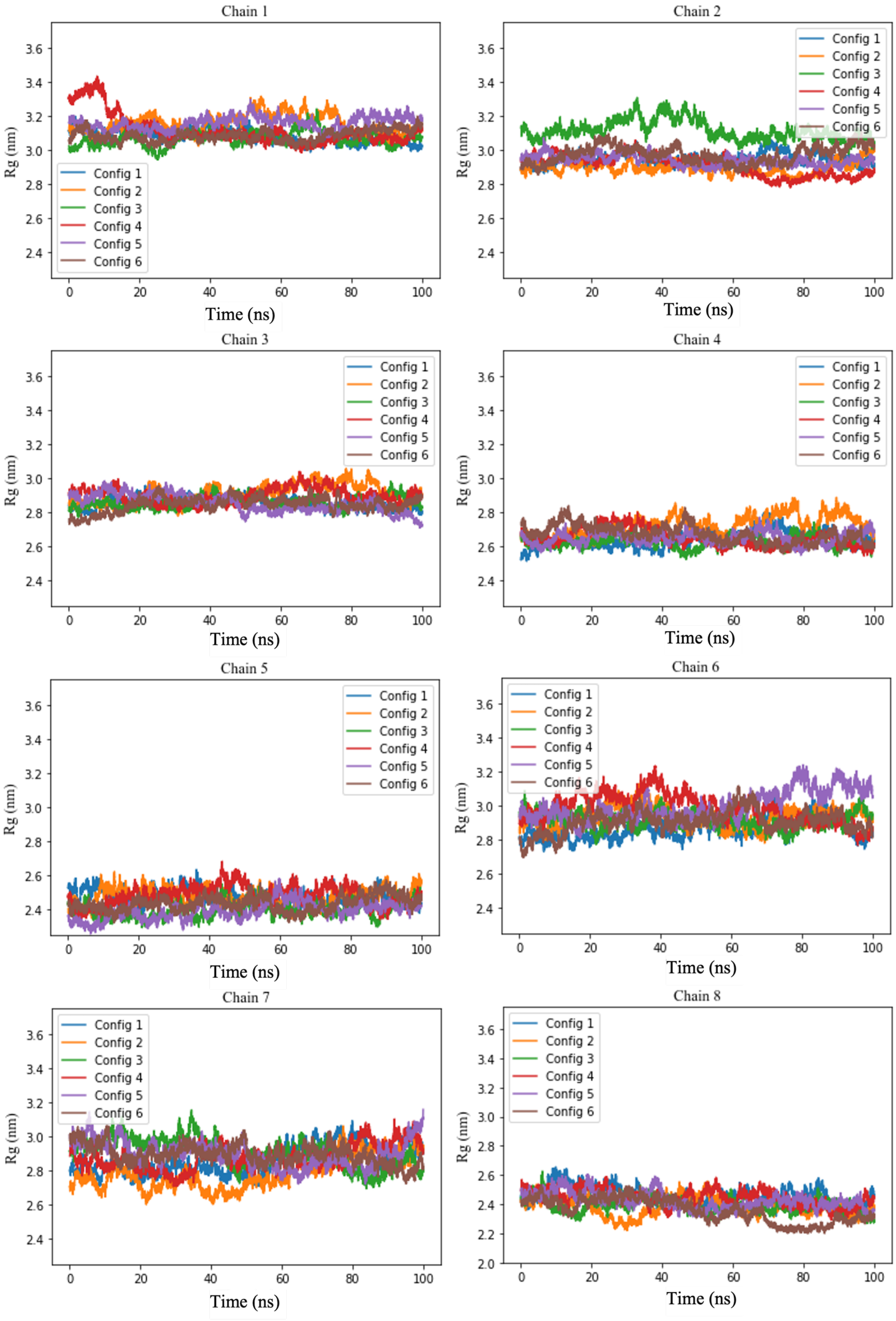

Figure S2. Time evolution of radius of gyrations (nm) for the micelles formed by chains 1-8 for six simulations with starting configurations for each chain type.

Probabily Density Distributions of Asphericity

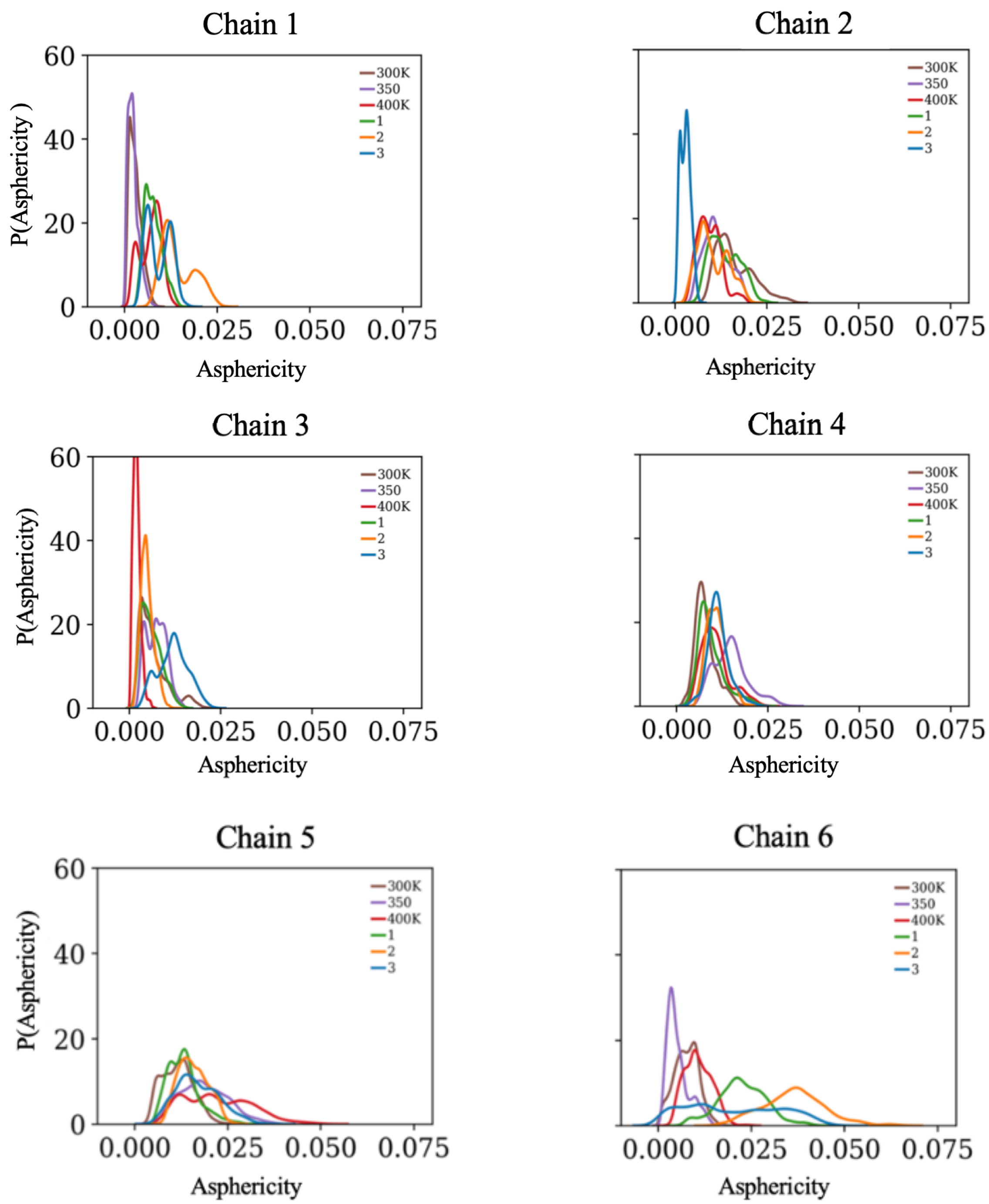

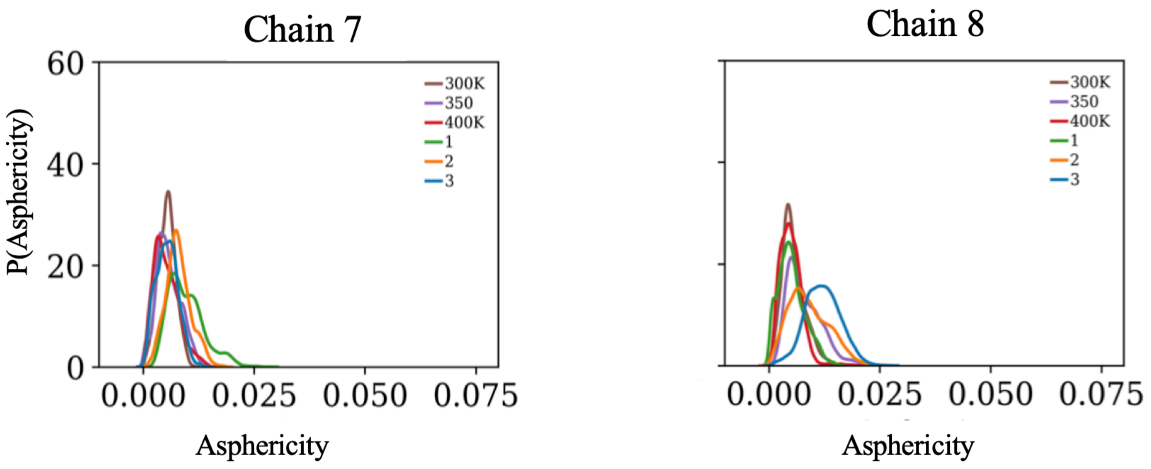

Figure S3. The probability density function (P) of asphericity from each of the six simulations resulting from six different initial starting configurations for the polypeptoid micelles formed by chain 1-8. Note that the kernel density estimation KDE was used to smooth the data.[1, 2]

Violin Plots: Additional Information

A violin plot visualizes the distributions of numeric data and its probability density. Furthermore, a violin plot is a combination of density plot (kernel density approximation) and a box plot containing information such as the median (white dot) and interquartile range (rectangle box), minimum value (lower tip), maximum value (upper tip). The width of each curve corresponds with the frequency of data points in each distribution. Multimodal distribution of data can be easily visualized using this graph. Two example violin plots are shown in Figure S2 to visualize the distributions of two different variables (Variable 1 and Variable 2). From the two example violin plots, the distribution of Variable 1 is narrower than Variable 2. It can be observed that Variable 1 is a unimodal distribution with single prominent peak while Variable 2 shows broader distribution with multiple peaks which corresponds to the multiple possibilities corresponding to the value of

Variable 2. All violin plots were plotted using the Seaborn library[3] in Python3[4] using the Kernel Denisty Approximation[2,1] to smooth the data.

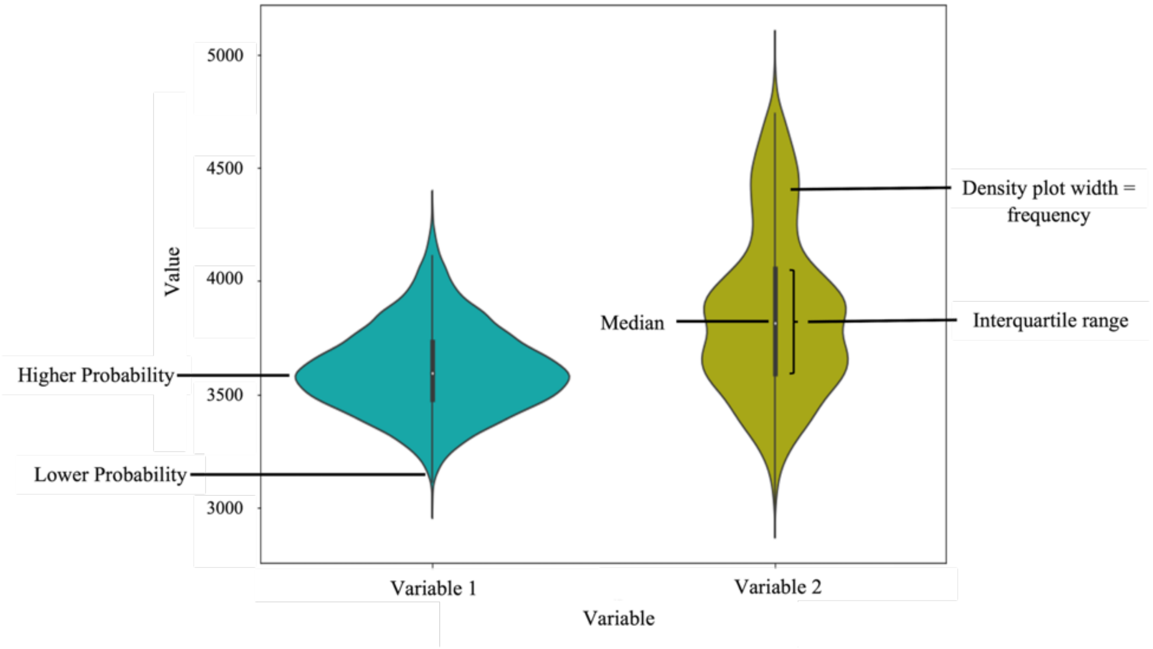

Figure S4. Example violin plot. Note that the kernel density estimation (KDE) was used to smooth the data for the representative violin plot.[1,2]

Additonal Data for Radius Gyration

Table S1. Experimental radius of gyration (nm), computed radius of gyration (nm) from MD simulations excluding the water molecules, computed radius of gyration (nm) including water molecules within 0.35 nm, computed scattering length weighted radius of gyration (Rg_b) excluding surrounding solvent, computed scattering length weighted radius

of gyration (Rg_b) including solvated D_2O within 0.35 nm, and the experimentally determined aggregation numbers (N) for micelles formed from chains 1-8.

Chain #	Experiment Rg_b	MD Rg	MD Rg (including solvated water molecules)	MD Rg_b	MD Rg_b (including solvated D_2O)	N
1	4.02 ± 0.04	3.11 ± 0.06	3.46 ± 0.08	3.36 ± 0.08	3.74 ± 0.08	28
2	3.70 ± 0.07	2.95 ± 0.08	3.29 ± 0.09	3.15 ± 0.11	3.53 ± 0.10	25
3	3.56 ± 0.05	2.87 ± 0.06	3.19 ± 0.07	3.08 ± 0.07	3.45 ± 0.08	23
4	3.24 ± 0.05	2.66 ± 0.06	2.96 ± 0.07	2.86 ± 0.08	3.19 ± 0.08	18
5	2.88 ± 0.03	2.45 ± 0.05	2.74 ± 0.06	2.61 ± 0.06	2.94 ± 0.06	13
6	3.19 ± 0.03	2.94 ± 0.14	3.30 ± 0.10	3.26 ± 0.09	3.58 ± 0.09	18
7	3.15 ± 0.03	2.88 ± 0.14	3.24 ± 0.09	3.18 ± 0.09	3.48 ± 0.09	17
8	2.88 ± 0.04	2.38 ± 0.10	2.68 ± 0.08	2.60 ± 0.07	2.88 ± 0.08	12

Radius Gyration Violin Plots (Excluding Water Molecules)

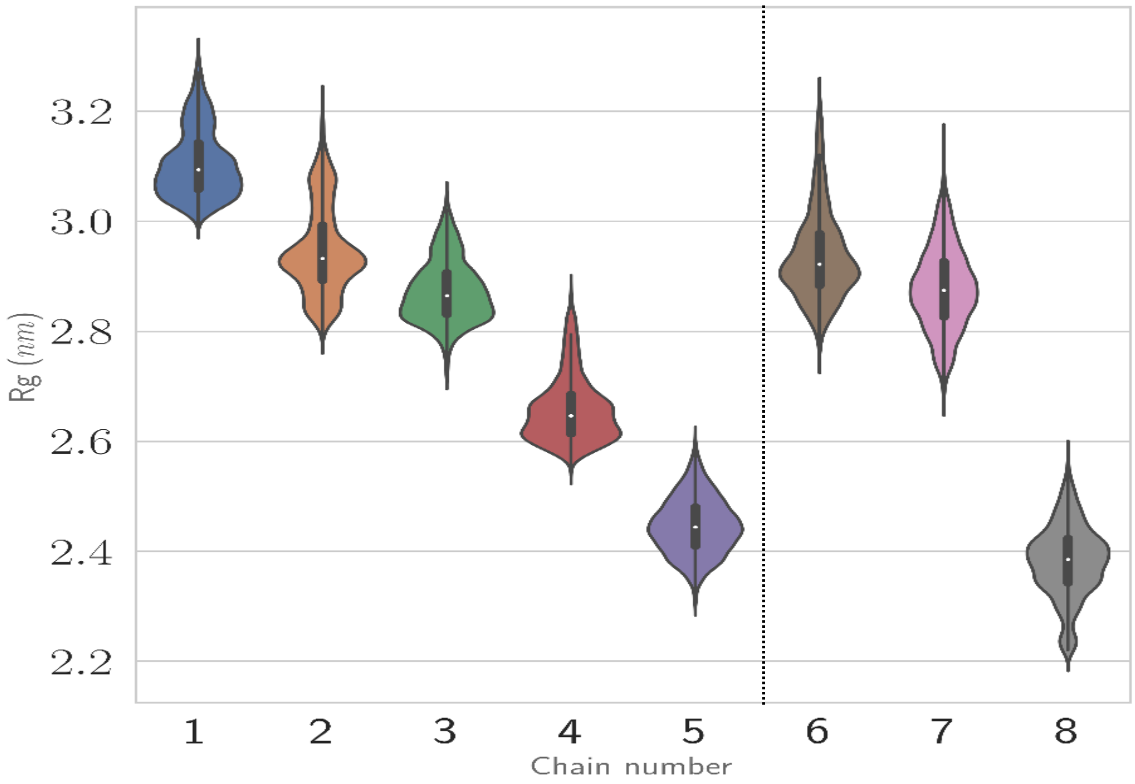

Figure S5. Radius of gyration (Rg) of the micelles formed by chain 1 to 8 (not including water). Note that the Kernel Density Estimation (KDE) was used to smoothen the data for the representative violin plot.[2,1]

Violin Plots of Radius Gyration Normalized per Surfactant Chain

In addition, radius of gyration has been normalized per polypeptoid surfactant chain by dividing the radius of gyration by the simulated aggregation numbers for each polypeptoid micelle (Figure S5).

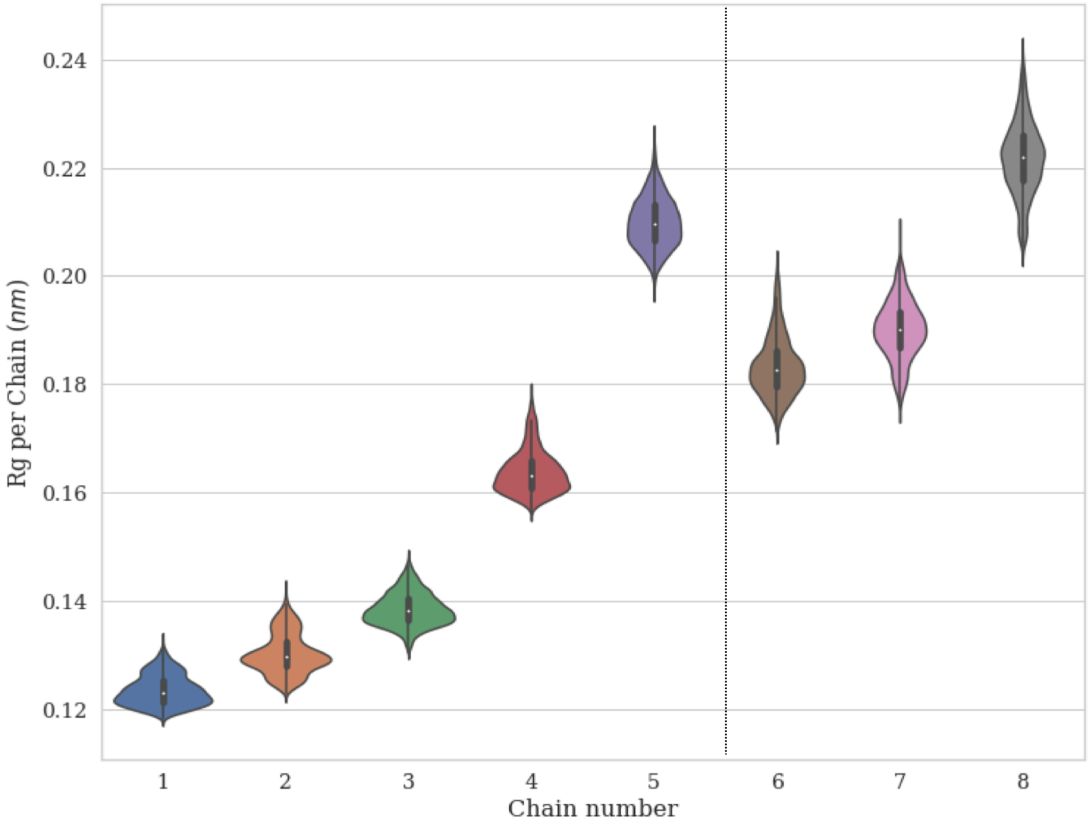

Figure S6. Violin plots of the radius of gyration (nm) that has been normalized per polypeptoid surfactant chain.

2D Joint Plots

(a)

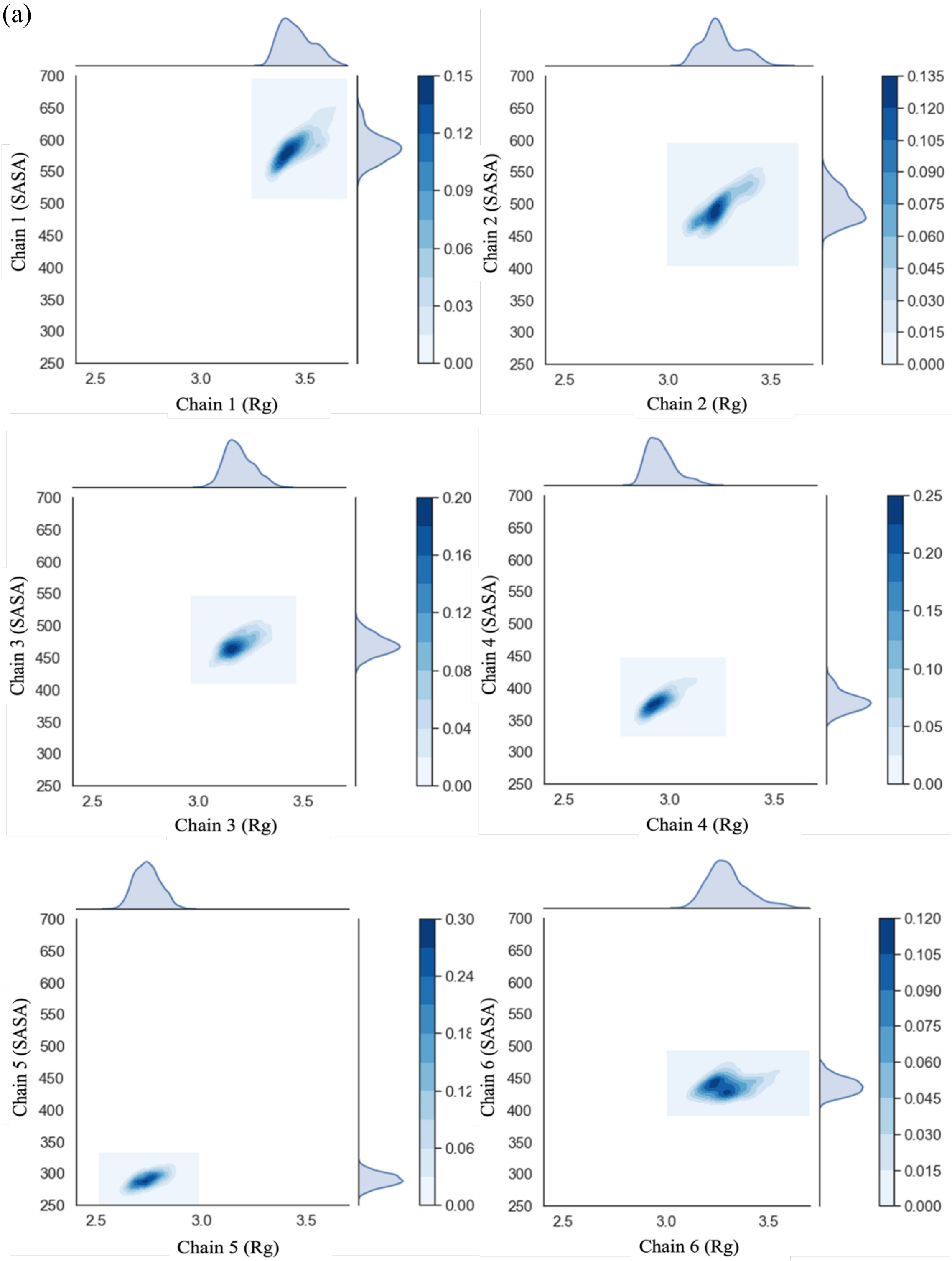

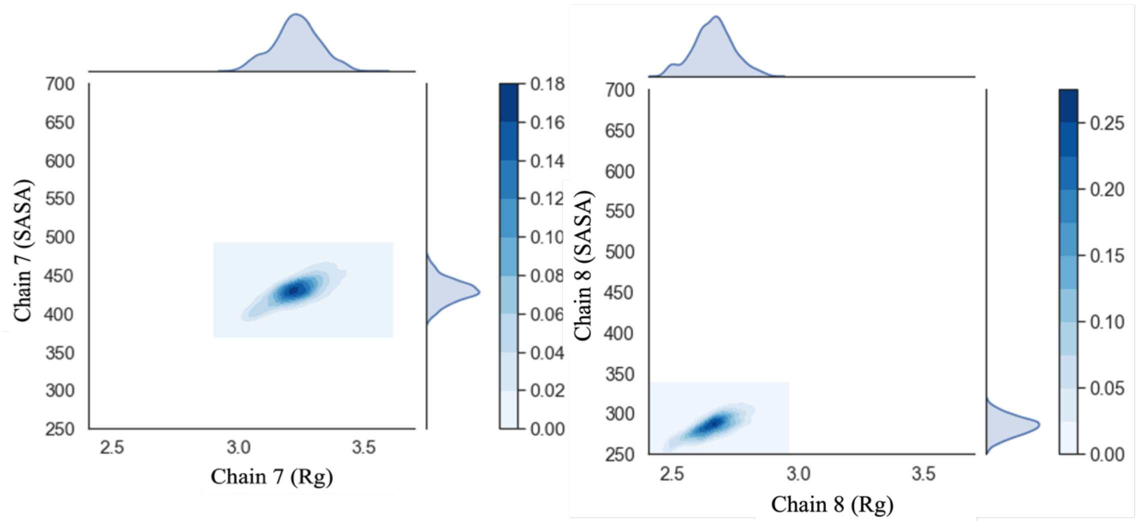

Chain 7 (SASA)
Chain 7 (Rg)
700
650
600
550
500
450
400
350
300
250
2.5
3.0
3.5
0.18
0.16
0.14
0.12
0.10
0.08
0.06
0.04
0.02
0.00
Chain 8 (SASA)
Chain 8 (Rg)
0.25
0.20
0.15
0.10
0.05
0.00

(b)

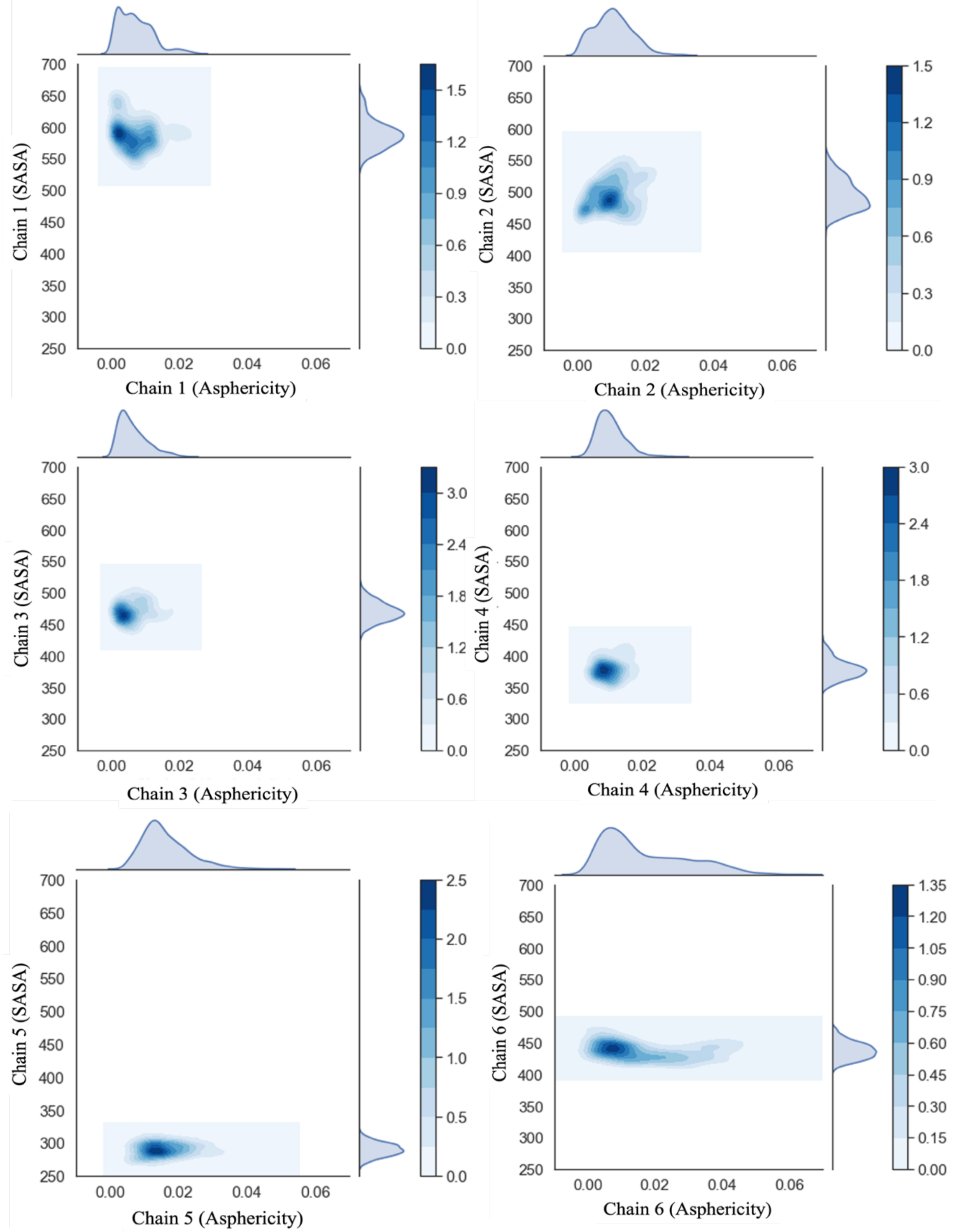
Chain 1 (SASA)
Chain 1 (Asphericity)
700
650
600
550
500
450
400
350
300
250
0.00
0.02
0.04
0.06
1.5
1.2
0.9
0.6
0.3
0.0
Chain 2 (SASA)
Chain 2 (Asphericity)
1.5
1.2
0.9
0.6
0.3
0.0
Chain 3 (SASA)
Chain 3 (Asphericity)
3.0
2.4
1.8
1.2
0.6
0.0
Chain 4 (SASA)
Chain 4 (Asphericity)
3.0
2.4
1.8
1.2
0.6
0.0
Chain 5 (SASA)
Chain 5 (Asphericity)
2.5
2.0
1.5
1.0
0.5
0.0
Chain 6 (SASA)
Chain 6 (Asphericity)
1.35
1.20
1.05
0.90
0.75
0.60
0.45
0.30
0.15
0.00

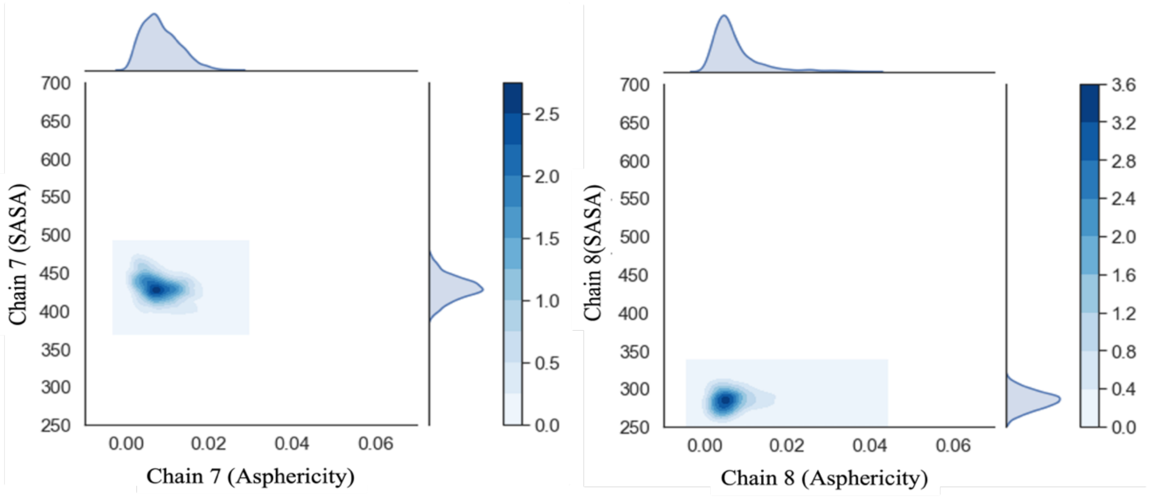
Chain 7 (SASA)
Chain 7 (Asphericity)
Chain 8(SASA)
Chain 8 (Asphericity)
700
650
600
550
500
450
400
350
300
250
0.00
0.02
0.04
0.06
2.5
2.0
1.5
1.0
0.5
0.0
3.6
3.2
2.8
2.4
2.0
1.6
1.2
0.8
0.4
0.0

(c)

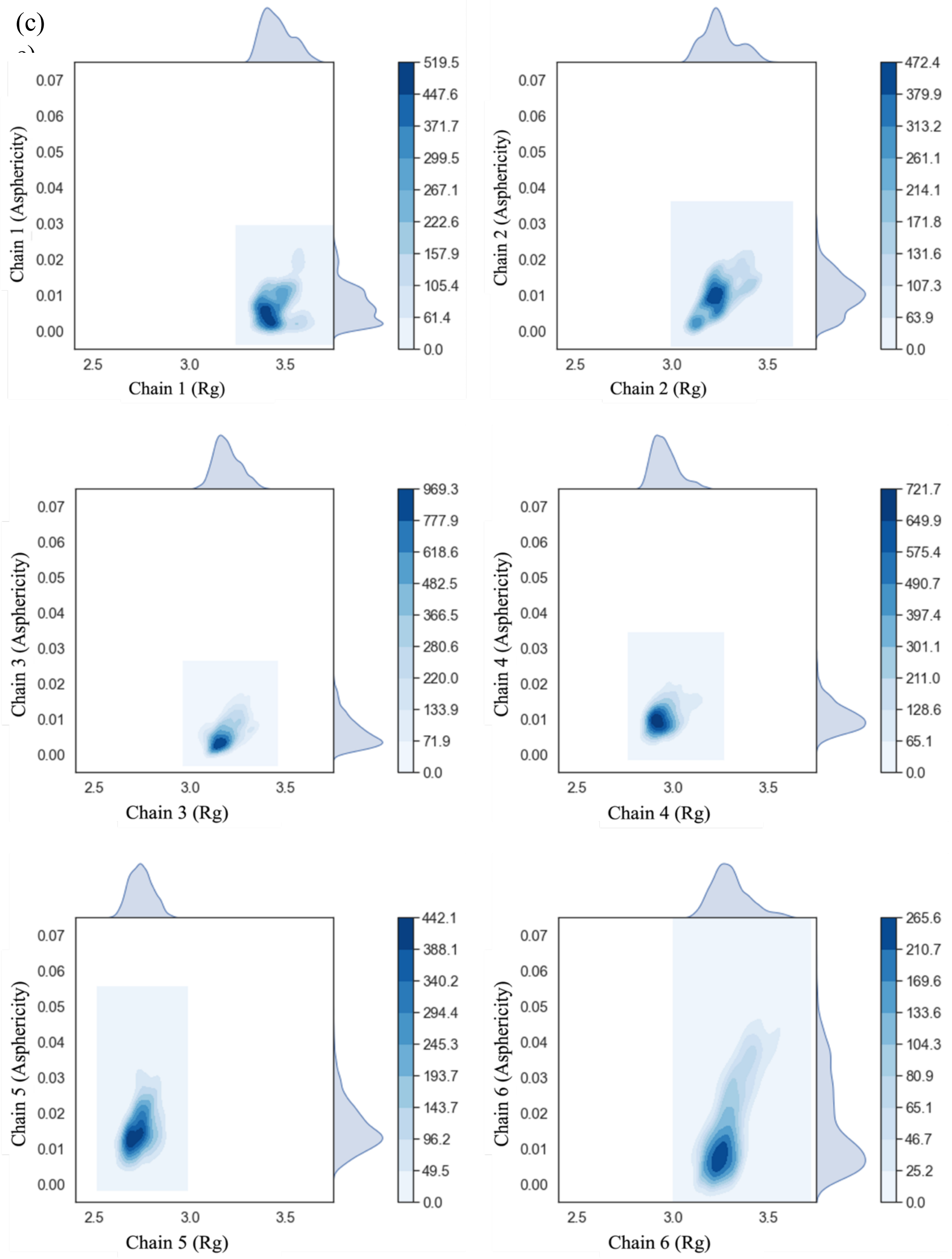
Chain 1 (Asphericity)
Chain 1 (Rg)
519.5
447.6
371.7
299.5
267.1
222.6
157.9
105.4
61.4
0.0
Chain 2 (Asphericity)
Chain 2 (Rg)
472.4
379.9
313.2
261.1
214.1
171.8
131.6
107.3
63.9
0.0
Chain 3 (Asphericity)
Chain 3 (Rg)
969.3
777.9
618.6
482.5
366.5
280.6
220.0
133.9
71.9
0.0
Chain 4 (Asphericity)
Chain 4 (Rg)
721.7
649.9
575.4
490.7
397.4
301.1
211.0
128.6
65.1
0.0
Chain 5 (Asphericity)
Chain 5 (Rg)
442.1
388.1
340.2
294.4
245.3
193.7
143.7
96.2
49.5
0.0
Chain 6 (Asphericity)
Chain 6 (Rg)
265.6
210.7
169.6
133.6
104.3
80.9
65.1
46.7
25.2
0.0
0.07
0.06
0.05
0.04
0.03
0.02
0.01
0.00
2.5
3.0
3.5

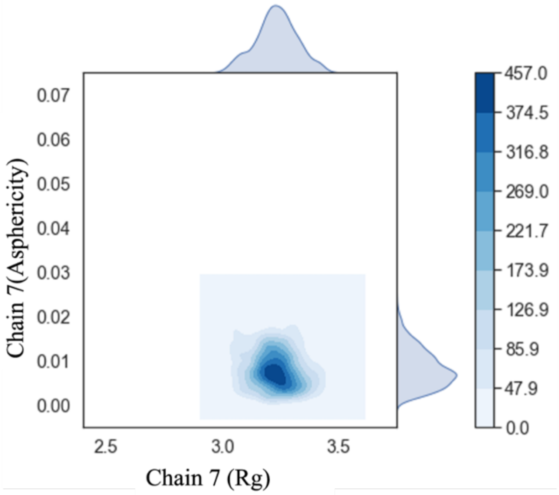
Chain 7(Asphericity)
0.07
0.06
0.05
0.04
0.03
0.02
0.01
0.00
2.5
3.0
3.5
Chain 7 (Rg)
457.0
374.5
316.8
269.0
221.7
173.9
126.9
85.9
47.9
0.0

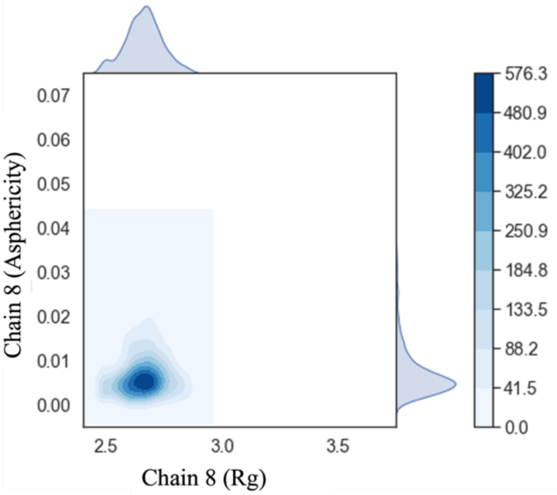
Chain 8 (Asphericity)
0.07
0.06
0.05
0.04
0.03
0.02
0.01
0.00
2.5
3.0
3.5
Chain 8 (Rg)
576.3
480.9
402.0
325.2
250.9
184.8
133.5
88.2
41.5
0.0

(d)

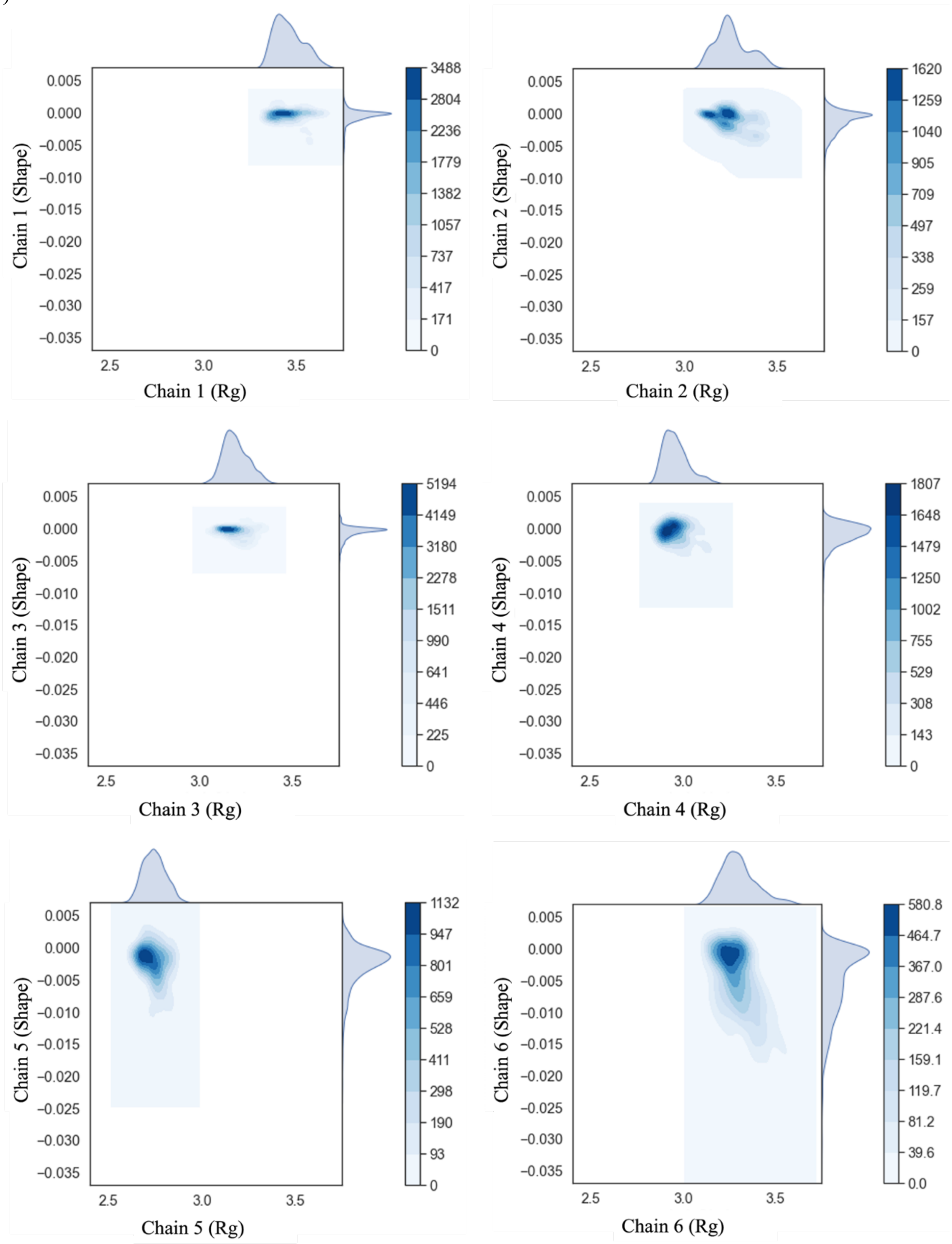
Chain 1 (Shape)
Chain 1 (Rg)
0.005
0.000
−0.005
−0.010
−0.015
−0.020
−0.025
−0.030
−0.035
2.5
3.0
3.5
3488
2804
2236
1779
1382
1057
737
417
171
0
Chain 2 (Shape)
Chain 2 (Rg)
1620
1259
1040
905
709
497
338
259
157
0
Chain 3 (Shape)
Chain 3 (Rg)
5194
4149
3180
2278
1511
990
641
446
225
0
Chain 4 (Shape)
Chain 4 (Rg)
1807
1648
1479
1250
1002
755
529
308
143
0
Chain 5 (Shape)
Chain 5 (Rg)
1132
947
801
659
528
411
298
190
93
0
Chain 6 (Shape)
Chain 6 (Rg)
580.8
464.7
367.0
287.6
221.4
159.1
119.7
81.2
39.6
0.0

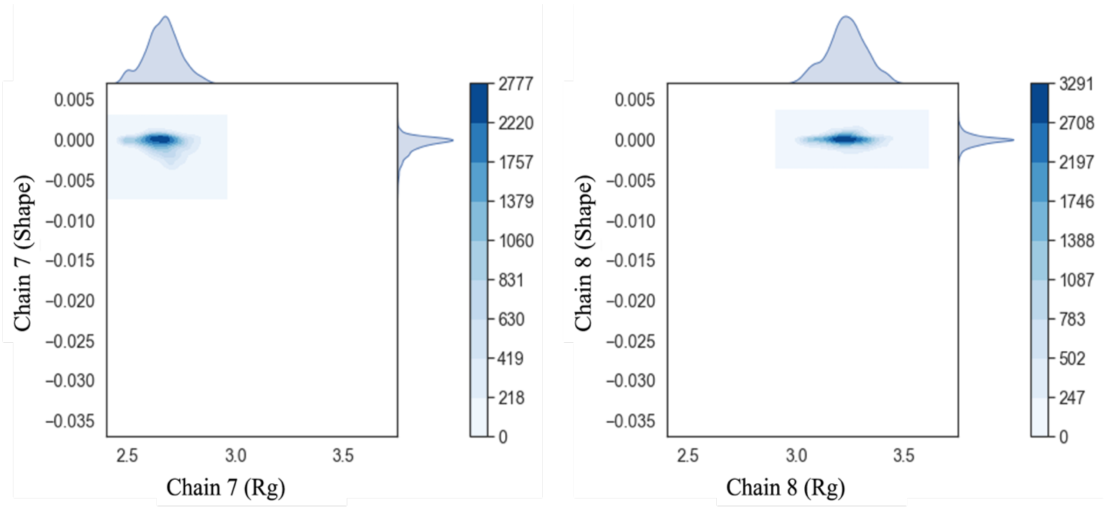

Figure S7. (a) 2D joint plots of SASA (nm^2) vs. Radius of Gyration (nm). (b) 2D joint plots of SASA (nm^2) vs. asphericity. (c) 2D joint plots of Aspherisity vs. Radius of Gyration (nm). (d) 2D joint plots of Shape vs. Radius of Gyration (nm). Note that the Kernel Density Estimation (KDE) was used to smoothen the data for the representative 2D plots.[2,1]

Pearson Correlation Coefficients

The Pearson correlation coefficients were calculated to measure the extent of the linear relationship between various shape descriptors in attempt to expose and make the explanation potential relationships between the various shape descriptors (i.e., asphericity vs. solvent accessible surface area (SASA), radius of gyration (Rg) vs. asphericity, Rg vs. shape, asphericity vs. shape, and shape vs. SASA).

Table S2. Summary of the calculated Pearson coefficients of the various shape descriptors comparing Rg vs asphericity, Rg vs. shape, and Rg vs. SASA.

**Chain no.**	**Rg vs. Asphericity**	**Rg vs. Shape**	**Rg vs. SASA**
1	0.271	-0.182	0.781
2	0.655	-0.461	0.833
3	0.510	-0.172	0.635
4	0.399	-0.264	0.760
5	0.404	-0.288	0.600
6	0.732	-0.548	0.311
7	-0.139	0.016	0.728
8	0.090	-0.279	0.722

Radial Distribution of Sodium (Na^+)-Oxygen (COO^-)

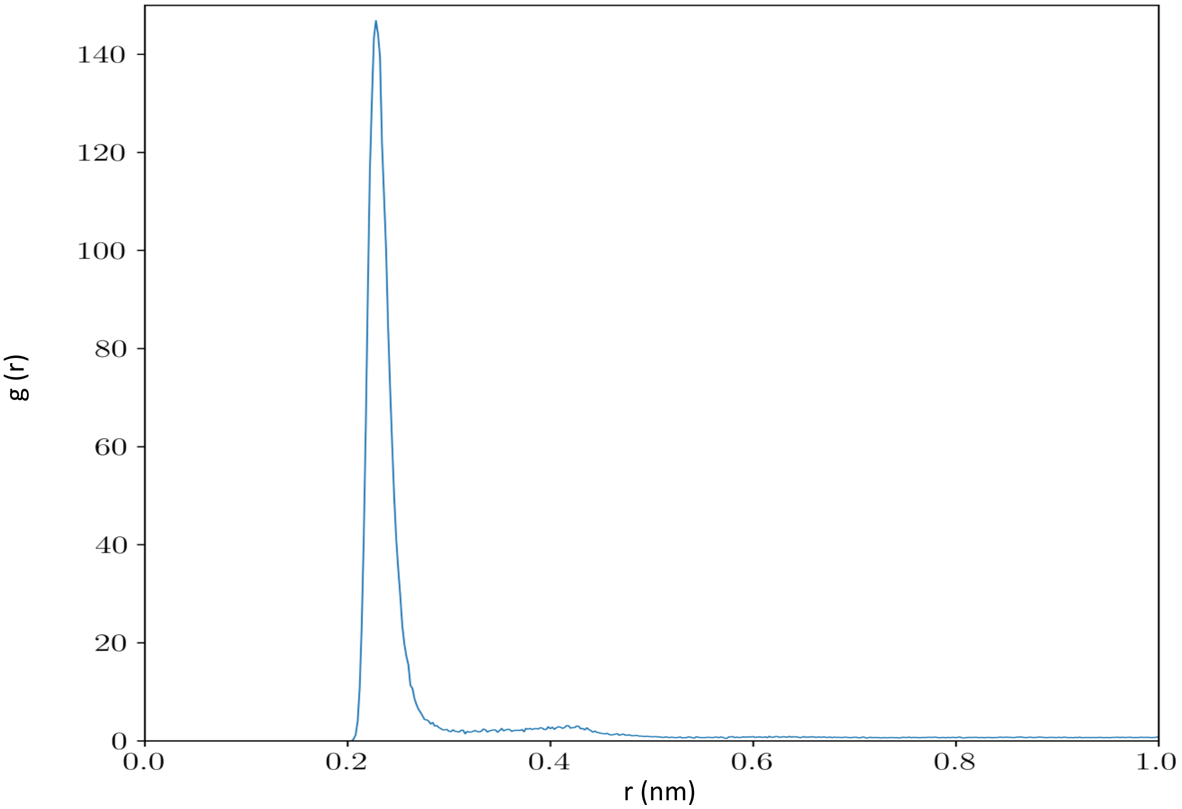

Figure S8. Representative radial distribution function of the sodium(Na^+)-oxygen (COO^-) calculated from the micelle formed by chain 6.

Radial Distribution Function of Oxygen (water)-Oxygen (COO^-)

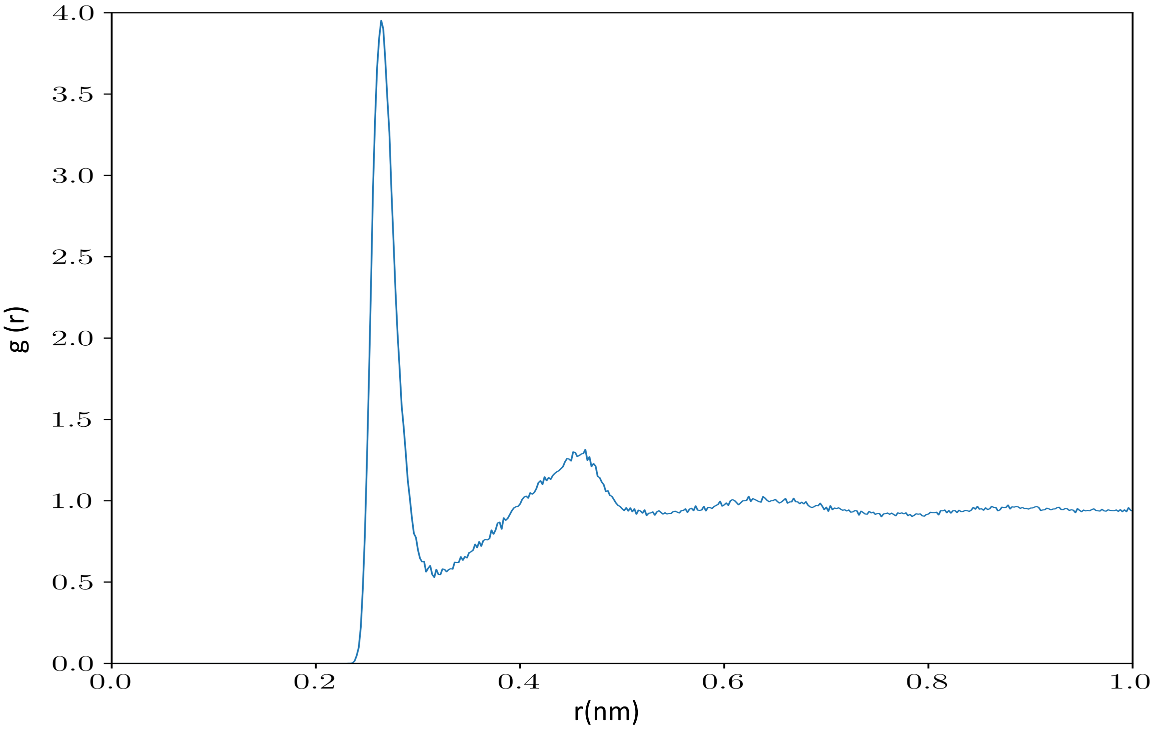

Figure S9. Representative radial distribution function of the oxygen (water)-oxygen (COO^-) calculated from the micelle formed by chain 6 .

References

(1) Murray, R. Remarks on Some Nonparametric Estimates of a Density Function. *The Annals of Mathematical Statistics* **1956**, *27* (3), 832-837. DOI: 10.1214/aoms/1177728190.

(2) Emanuel, P. On Estimation of a Probability Density Function and Mode. *The Annals of Mathematical Statistics* **1962**, *33* (3), 1065-1076. DOI: 10.1214/aoms/1177704472.

(3) Waskom, M. L. Seaborn: Statistical Data Visualization. *J. Open Source Softw.* **2021**, *6*, 3021.

(4) Van Rossum, G. D., Fred. *Python 3 Reference Manual*; CreateSpace, 2009. DOI: 10.5555/1593511.
